# A Systematic Review and Meta-Analysis of Executive Function Outcomes in Pediatric Central Nervous System Tumor Survivors

**DOI:** 10.62641/aep.v54i1.2053

**Published:** 2026-02-15

**Authors:** Esperanza Bausela Herreras

**Affiliations:** ^1^Department of Health Sciences, Public University of Navarra, Campus de Arrosadia s/n, 31006 Pamplona, Comunidad Foral de Navarra, Spain

**Keywords:** astrocytoma, BRIEF, executive function, neurofibromatosis-1, medulloblastoma, pilocytic astrocytoma

## Abstract

**Background:**

This review aimed to determine whether executive dysfunction is a characteristic of survivors of central nervous system tumors in children and adolescents, including Astrocytoma, Neurofibromatosis-1, Medulloblastoma, and Pilocytic Astrocytoma.

**Methods:**

A review and meta-analysis of executive function assessed with Behavior Rating Inventory of Executive Function (BRIEF) in individuals with these tumor types.

**Results:**

The main findings of the meta-analyses can be summarized as follows: (i) Neurofibromatosis type 1 (NF1) – BRIEF (parents): Children with NF1 show significant deficits in executive functions according to the parent-rated BRIEF, with an overall model effect size of d = 0.81 (*p* < 0.001). The most affected areas are working memory, monitoring, and metacognition, indicating that these deficits are consistent and clinically relevant. (ii) NF1 – BRIEF-P (parents and teachers): In this meta-analysis, the overall model effect size was d = 0.37 (*p* < 0.001), showing moderate but significant difficulties in executive functions. Both parents and teachers report problems in working memory and emerging metacognition, reflecting a consistent pattern across different observational contexts. (iii) Medulloblastoma vs. other tumors: Patients with medulloblastoma exhibit marked deficits in executive functions compared to other brain tumors, with an overall model effect size of d = –0.74 (*p* < 0.001). The most affected areas include inhibition, initiation, regulation, and metacognition, with consistent findings across the included studies.

**Conclusions:**

Executive deficits are observed in individuals with brain tumors or survivors, significantly affecting their academic, social, and emotional lives. Early identification, along with educational and neuropsychological support, is essential to preventing these deficits from interfering with academic, personal, and professional functioning.

## Introduction

Survivors of Central Nervous System (CNS) tumors face a significant risk of long-term complications affecting physical, motor, and cognitive domains [1]. Among these sequelae, executive function (EF) deficits are particularly common and may arise both as a direct consequence of the disease and as a result of oncological treatments such as chemotherapy and radiotherapy [2, 3, 4]. In particular, cranial radiation therapy, especially craniospinal irradiation, has been associated with more severe executive dysfunction compared with surgery or focal therapies [5, 6].

Moreover, the extent of executive impairment is influenced by tumor characteristics, treatment type, and concomitant psychological factors such as depression and anxiety [7]. Tumor location also plays a key role: infratentorial (cerebellar) tumors primarily affect inhibition, set-shifting, and planning, while supratentorial tumors tend to impact working memory and verbal reasoning [8]. Additional risk factors include younger age at diagnosis, presence of hydrocephalus, and complex or multiple treatment regimens [5, 9].

Beyond treatment-related effects, certain clinical conditions present distinct profiles of executive dysfunction. In neurofibromatosis type 1 (NF1), genetic alterations affecting neurofibromin lead not only to physical manifestations but also to deficits in planning, inhibitory control, attention, and working memory [10]. Similarly, astrocytomas, particularly low-grade tumors, are associated with persistent difficulties in attention, planning, and cognitive control even after surgical treatment, suggesting a tumor-related origin of executive impairment [11, 12, 13, 14].

Medulloblastoma, a common pediatric brain tumor, causes significant EF impairments linked to white matter and frontal lobe damage caused by both the tumor and treatments such as radiotherapy [11, 15, 16].

Even in pilocytic astrocytoma (PA), where general intelligence is often preserved, survivors show notable executive deficits—particularly in sustained attention, processing speed, and visuospatial memory—that affect daily functioning and academic performance [11].

Overall, across these conditions, executive dysfunction emerges as a widespread and multifactorial consequence of CNS tumors and their treatments, highlighting the need for targeted cognitive rehabilitation strategies to optimize long-term functional outcomes [17, 18].

EF deficits in survivors of pediatric CNS tumors significantly affect daily life and long-term outcomes. Survivors often exhibit impairments in working memory, inhibition, planning, cognitive flexibility, self-regulation, and attention [5, 8, 19, 20, 21]. These deficits are found across tumor types and treatment modalities, persist for years, and may even worsen over time [5, 6, 20]. Table 1 (Ref. [5, 8, 19, 20, 21]) presents key findings on executive function impairments in children surviving CNS tumors.

**Table 1.** **Executive function deficits in pediatric survivors of Central Nervous System (CNS) (own elaboration)**.

Type of deficit	Risk factors	References
Working Memory	Radiation, tumor location	[8, 20]
Inhibition/Planning	Infratentorial tumors, radiation	[8, 21]
Attention	Radiation, age at diagnosis	[5, 20]
Metacognition	Early age at diagnosis	[19]

Note: CNS, Central Nervous System.

These deficits have a significant impact on quality of life, being associated with lower health-related well-being, reduced social skills, and impaired adaptive functioning [19, 21, 22]. They can also hinder academic achievement, employment, and independence in adulthood [6].

In pediatric cancer survivors — particularly those treated for brain tumors — major executive function impairments have been reported, especially through parent-rated assessments using the Behavior Rating Inventory of Executive Function (BRIEF) [23]. While inhibitory control often remains relatively preserved, difficulties in cognitive flexibility, information updating, and working memory are frequently observed [4, 23].

In long-term adult survivors of CNS tumors, deficits in processing speed, attention, and working memory have also been identified, impacting executive control. Neuropsychological assessment is essential to detect deficits before and after treatment. Both parent-rated and performance-based measures consistently reveal widespread executive dysfunction with negative effects on everyday functioning [21].

Several studies highlight that both cancer and its treatments adversely affect executive functions, and the BRIEF is a useful tool to identify executive difficulties in daily life contexts [24]. However, BRIEF scores do not always correlate with performance-based neuropsychological measures, suggesting that they may capture different aspects of executive function [23]. Both the parent and teacher versions of BRIEF provide valuable information, although the teacher form tends to align more closely with direct EF task performance.

Research on this topic has evolved from 2008 to 2024, primarily addressing executive syndromes and frontal localization, treatment impacts on brain networks, and strategies for assessment and rehabilitation [7, 25, 26, 27, 28, 29, 30, 31, 32].

In summary, executive function deficits are a frequent and lasting consequence of pediatric CNS tumors, significantly impacting daily life and long-term outcomes. The risk is higher in patients treated with cranial radiotherapy, those with infratentorial tumors, or those diagnosed at an early age. Early detection and targeted interventions are crucial, as executive dysfunction is multifactorial, resulting from direct damage, disruption of brain networks, and treatment effects. Comprehensive rehabilitation and ongoing assessment are essential to improve patients’ quality of life and functional recovery [5].

### Research Objectives

This review aims to synthesize the available evidence on executive function deficits in pediatric CNS tumor survivors, identify associated risk factors, and provide useful information for evaluation, rehabilitation, and clinical follow-up strategies.

This review aims to examine executive function deficits in individuals diagnosed with NF1, Astrocytoma, Medulloblastoma, and Pilocytic Astrocytoma, focusing specifically on studies using the BRIEF scales in their different versions [33, 34]. Focusing solely on BRIEF-based studies allows for an ecologically valid assessment of executive functions and ensures consistency across research.

It is expected that individuals with these diagnoses will exhibit greater executive function impairments than the normative population, reflected in higher BRIEF scores. Deficits are anticipated to be particularly pronounced in subscales measuring inhibition, cognitive flexibility, and working memory, as these domains are most commonly affected in pediatric brain tumor populations.

## Materials and Methods

We chose to focus solely on BRIEF studies to enable an ecological evaluation of executive functions, ensuring consistency across the research. Individuals diagnosed with NF1, Astrocytoma, Medulloblastoma, and Pilocytic Astrocytoma will show a significantly greater degree of executive function impairment than those in the normative population.

### Eligibility Criteria for Studies and Selection Process 

Executive functions are cognitive processes essential for self-regulation and academic performance. Survivors of childhood CNS tumors frequently exhibit deficits in these functions as a consequence of the tumor and its treatments. However, the magnitude and consistency of these impairments remain under debate.

To synthesize the available evidence, the following research question was formulated according to the PICOS framework (Population, Intervention, Comparison, Outcomes, and Study Design) was used [35]:

(i) P (Population/Participants): Children and adolescents (≤18 years) who are survivors of or diagnosed with central nervous system tumors, including Astrocytoma, Medulloblastoma, Pilocytic Astrocytoma, and NF1.

(ii) I (Intervention/Exposure): Presence or history of a childhood brain tumor and its impact on executive functioning after treatment or during survivorship.

(iii) C (Comparison): Children and adolescents without a history of brain tumors (healthy control group or matched by age and educational level).

(iv) O (Outcomes): Executive functioning assessed using the BRIEF, considering total scores and subscales.

(v) S (Study design): Observational studies (cross-sectional, cohort, case-control), systematic reviews, and meta-analyses reporting quantitative results.

The review will include studies in which children and adolescents diagnosed with CNS tumors (Neurofibromatosis type 1, Medulloblastoma, Astrocytoma and Pilocytic Astrocytoma) were exposed to any standard oncological treatment (surgery, chemotherapy or radiotherapy) during pediatric age. Only studies that assessed executive functioning using the BRIEF scales in any version or translation will be considered. Both cross-sectional and longitudinal designs, including descriptive or comparative-causal (ex post facto) studies, are eligible. Studies must report sufficient quantitative data to allow calculation of effect sizes for the meta-analysis.

The review will exclude studies that meet any of the following criterio:

(i) Age at diagnosis/exposure: Patients diagnosed after 18 years of age, to focus the study on the pediatric population and individuals exposed in utero.

(ii) Type of diagnosis: Secondary or metastatic tumors and tumors unrelated to the CNS, such as leukemia or sarcoma, which present different neurocognitive mechanisms.

(iii) Type of treatment: Participants treated only with surgery or therapies not directed at the CNS, whose inclusion could hinder the relationship between treatment and executive deficits.

(iv) Neuropsychological assessment: Studies that do not use the BRIEF, to ensure comparability and methodological consistency.

(v) Study design and clinical status: Case studies, reviews, qualitative research, unpublished conference abstracts, patients under active treatment, or those evaluated less than 6 months post-treatment are excluded, ensuring data are reliable and comparable.

(vi) Data availability: Studies without sufficient quantitative information for statistical analysis or meta-analysis.

The present work includes the following inclusion and exclusion criteria for review studies (see Table 2).

**Table 2.** **Inclusion and exclusion criteria for review studies (own elaboration)**.

Domain	Inclusion criteria	Exclusion criteria
Age at Diagnosis/Exposure	Individuals under 18 years of age (both sexes) at the time of diagnosis of a primary brain tumor, or exposed in utero to oncological treatments with potential effects on the CNS.	Individuals diagnosed after 18 years of age.
Type of Diagnosis/Exposure	Patients diagnosed with primary brain tumors (e.g., medulloblastoma, astrocytoma, glioma, ependymoma) confirmed by neuroimaging and/or histopathological examination.	Patients with secondary or metastatic brain tumors, or with non-CNS-related tumors (e.g., leukemia, lymphoma, sarcoma).
Type of Treatment	Participants who received radiotherapy, chemotherapy, or concurrent chemoradiotherapy, provided that CNS exposure is documented in the medical record.	Participants treated only with surgery or with non-CNS-directed therapies (e.g., peripheral radiotherapy).
Neuropsychological Assessment	Studies assessing executive functioning using BRIEF in any of its validated versions or translations, reporting at least one global (GEC) or specific index (BRI, MI).	Studies using other instruments (e.g., WCST, Stroop, TMT) or not reporting BRIEF-derived outcomes.
Study Design	Ex post facto (descriptive, comparative-causal) quantitative designs published in peer-reviewed journals in English or Spanish.	Case studies, reviews, qualitative designs, conference abstracts, or unpublished theses.
Clinical Status at the Time of Evaluation	Participants in remission or stable disease phase, evaluated at least 6 months post-treatment.	Participants under active oncological treatment or evaluated within 6 months after treatment.
Data Availability	Studies providing quantitative data (mean, standard deviation, or effect size) for BRIEF measures.	Studies not reporting sufficient data for statistical analysis or meta-analysis.

Note: BRI, Behavioral Regulation Index; CNS, Central Nervous System; GEC, Global Executive Composite; MI, Metacognition Index; Stroop, Stroop Color and Word Test; TMT, Trail Making Test; WCST, Wisconsin Card Sorting Test.

### Search Strategy

The search strategy adheres to the principles outlined in the PRISMA statement [36]. For this purpose, the databases PubMed, Springer Link, and Scopus were accessed, choosing articles released between 2010 and March 2024.

The year 2010 was selected as the starting point taking into account the publication timeline of the BRIEF and its standardized versions. The original BRIEF (informant version, 2000; self-report, 2004) and the BRIEF-P (2003) were followed by the BRIEF-A (2004) and later by the revised BRIEF-2 (2015), which has become the most widely used instrument in pediatric neuropsychological research. Therefore, limiting the search to studies published from 2010 onward ensures that the included studies reflect the modern versions and standardized application of the BRIEF scales.

Moreover, from 2010 onward, significant updates were implemented in both neurological and oncological diagnostic criteria for example, the transition toward molecular classifications of CNS tumors and the WHO revisions in 2016 and 2021 as well as in pediatric oncology treatment protocols, which began to systematically incorporate cognitive preservation strategies and neuropsychological follow-up. Consequently, studies published since this date tend to show greater homogeneity in clinical criteria, neuroimaging techniques, and cognitive assessment methods, improving the methodological comparability and clinical relevance of the results integrated in the meta-analysis.

To begin with, an initial bibliographic search was performed using the keywords “cancer”, “tumor”, and “executive function” in English. Subsequently, the studies were classified according to the type of tumor, and those focusing on diagnoses such as NF1, Astrocytoma, Medulloblastoma, and Pilocytic Astrocytoma were chosen.

The complete search strategy used to identify the studies is presented in **Supplementary Material 1**, in order to ensure transparency and reproducibility of the review process.

### Included Studies 

An initial search yielded a total of 391 articles, allowing for an overview of how research in this field has evolved over time. Following this screening, a refined collection of 48 articles was compiled, enabling a detailed assessment of the progression of research on the topic and the identification of studies suitable for a subsequent meta-analysis.

Ultimately, 48 studies were selected to form the basis of this review. In **Supplementary Material 2**, a collection of 48 articles is presented, enabling the observation of the progression of research on the subject and the identification of studies suitable for a subsequent meta-analysis.

Of these, 8 studies were included in the meta-analysis with the following diagnosis: (i) NF1 [37, 38, 39, 40, 41, 42]; (ii) Astrocytoma [43]; (iii) Medulloblastoma and Pilocytic astrocytoma [11].

The meta-analysis included only 8 of the 48 selected studies, as only these provided complete and comparable quantitative data necessary for a reliable statistical analysis. The analysis was conducted considering previous studies that analyze profiles in clinical populations [44, 45]. The remaining studies met inclusion criteria but lacked sufficient data for calculating standardized effects, ensuring the validity and reliability of the meta-analytic results.

Through an initial screening process, studies whose titles indicated no relevance to the research objective were excluded. Subsequently, a second screening and suitability assessment were conducted by reviewing the abstracts, ultimately selecting those that met the established criteria.

The studies included in the meta-analysis (8 studies) are presented in Table 3 (Ref. [11, 37, 38, 39, 40, 41, 42, 43]).

**Table 3.** **Studies included in the meta-analysis (own elaboration)**.

No.	Study	Journal/Oncology-specific	Title (BRIEF)	Country	Sample (n)	Cancer	Age (diagnosis and/or assessment)	Sex N (SD)	Methodology	Instrument	Specific results	Overall results	Informant
1.	[37]	Journal of Clinical and Experimental Neuropsychology/ NO	NO	France	NF1 Group: (n = 33) Control Group: (n = 52) Informants: Parents Group: (n = 31) Teachers Group: (n = 18)	NF1	3–5 years NF1 Group: 56.67 (11.27) (months) Control Group: 55.75 (10.37) (months)	NF1 Group: 17/16 (male/female) Control Group: 27/25 (male/female)	IV: NF1 Group vs. Control Group DV: BRIEF-P WPPSI-IV	Comparati-ve-causal	BRIEF-P	Children with NF1 show early executive dysfunction, highlighting the importance of early and systematic assessment using complementary performance-based tests and questionnaires.	YES Informant: Parents Teachers
2.	[38]	Journal of Pediatric Psychology/NO	NO	United States of America	NF1 Group: (n = 26) Control Group: (n = 37)	NF1	NF1 4.53 (0.87) G. Control 4.51 (0.89)	NF1 Males: 17 (65%) Females: 9 (34%) Control Group Males: 23 (62%) Females: 14 (38%)	IV: NF1 Group vs. Control Group DV: BRIEF-P	Comparati-ve-causal	BRIEF-P	Working Memory (WM) emerged as an area of difficulty for young children with NF1.	YES Informant: Parents Teachers
3.	[41]	Neuropsychological Rehabilitation/ NO	NO	Israel	NF1 Group: (n = 29) Control Group: (n = 27)	NF1	NF1 Group 12.3 (2.6) G. Control 12.4 (2.5)	NF1 Males: 8 Females: 21 Control Group Males: 8 Females: 19	IV: NF1 Group vs. Control Group DV: BADS-C BRIEF – Parents ACES – Teacher	Comparati-ve-causal	BRIEF	Children with NF1 exhibit executive dysfunction, which partially accounts for their difficulties in academic achievement.	YES Informant: Parents
4.	[39]	The Journal of Pediatrics/ NO	NO	Australia	NF1 Group: (n = 43) Control Group: (n = 43)	NF1	NF1 Group 40.23 (0.72) months Control Group 40.16 (0.48) months	G. NF1 Males = 32 (74%) Females = 11 (26%) Control Group Males = 32 (74%) Females = 11 (26%)	IV: NF1 Group vs. Control Group DV: BASC-II BRIEF-Parents CADS-Parents	Comparati-ve-causal	BRIEF-P	The deficit in general intelligence and early cognitive difficulties in children with NF1 are detectable from preschool age and affect initial academic performance.	YES Informant: Parents
5.	[42]	Child Neuropsychology/NO	NO	Australia	Control Group: (n = 55) NF1 Group: (n = 191) NF1 Typical Group: (n = 41) NF1 Bordeline Group: (n = 30) NF1 Impaired Group: (n = 120)	NF1	Control 11.81 (2.61) NF1 10.38 (2.36) NF1 Typical 11.61 (2.75) NF1 Bordeline 9.98 (2.29) NF1 Impaired 10.06 (2.11)	Males = Control Group: 22 (40) NF1 Group: 104 (54, 45) NF1 Típical Group: 27 (65, 85) NF1 Límite: 13 (56, 67) NF1 Deteriorado: 64 (53, 33)	IV: NF1 Group vs. Control Group DV: RCFT, CI, Visuospatial skills, BRIEF, Tower of London CADS	Comparati-ve-causal	BRIEF	Most children with NF1 exhibited significant difficulties in visuospatial skills, poorer cognitive performance, and executive function deficits compared to their peers. Visuospatial deficits were the main factor affecting performance, while executive functions and age also had independent effects.	YES Informant: Parents
6.	[40]	Child Neuropsychology/NO	NO	Australia	NF1 Group: (n = 199) Control Group: (n = 55) Participants who were assessed with the BRIEF NF1 Group: (n = 168) Control Group: (n = 39)	NF1	6–16 NF1 Group: 10.62 (2.28) Control Group: 11.24 (2.03)	G. NF1: Males = 108 Females = 91 Control Group	IV: NF1 Group vs. Control Group DV: BRIEF, CADS, WISC-III, WISC-IV	Comparati-ve-causal	BRIEF (Parents and Teachers)	Children with NF1 show deficits in attention and executive functions, and cognitive and functional tests measure different aspects. It is recommended to complement neuropsychological assessment with functional tools to better guide intervention.	YES Informants: Parents
7.	[43]	Neuro-Oncology/YES	NO	United Kingdom	Medulloblastoma Group: (n = 32) Astrocytoma Group: (n = 34) Control Group: (n = 38)	Brain Tumor	8–14 years Cerebellar Group N: Medulloblastoma Assessment age: 10.2 (8–14) Diagnosis age: 10.4 (8–14) Astrocytoma Assessment age: 10.4 (8–14) Diagnosis age: 9.2 (5–14) Control Group Assessment age: 10.4 (8–14)	Cerebellar Group Medulloblastoma Group Females: 13 (41%) Astrocytoma Group Females: 23 (68%) Control Group Females: 19 (50%)	VI: Cerebellar Group Medulloblastoma Group Astrocytoma Group Control Group VD: IQ BRIEF – Parents and Teachers SDQ – Parents, Teachers, Child PedsQL – Parents and Child	Comparati-ve-causal	BRIEF	The PedsQL, BRIEF, and SDQ show moderate accuracy in detecting children with IQ ≤80; PedsQL is useful in clinical settings, while BRIEF and SDQ are suitable for educational settings.	YES Informants: Parents Teachers
8.	[11]	Applied Neuropsychology: Child/NO	NO	United States of America	Medulloblastoma Group: (n = 36) Pilocytic Astrocytoma Group: (n = 20)	Pediatric Brain Tumor: Medulloblas-toma Pilocytic Astrocytoma	Medulloblastoma Age at diagnosis: 8.55 (4.34) Age at assessment: 14.07 (3.45) Pilocytic Astrocytoma Age at diagnosis: 5.40 (4.34) Age at assessment: 12.84 (2.67)	Medulloblastoma Males: 24 (66.7%) Females: 12 (33.3%) Pilocytic Astrocytoma Males: 11 (55.0%) Females: 9 (45.0%)	VI: Type of cancer: Medulloblastoma Group Pilocytic Astrocytoma Group VD: BRIEF	Comparati-ve-causal	BRIEF	Pilocytic Astrocytoma survivors were rated as having poorer executive function than MB survivors, suggesting that parent questionnaires like the BRIEF reflect real-world difficulties and should be compared with performance-based measures.	YES Informants: Parents

Note: DV, Dependent Variable; IV, Independent Variable; NF1, Neurofibromatosis type 1; IQ, Intelligence Quotient Instruments: ACES, Assessment of Children’s Executive Skills; BADS-C, Behavioral Assessment of the Dysexecutive Syndrome for Children; BRIEF-P, Behavior Rating Inventory of Executive Function – Preschool version; CADS, Conners’ ADHD Diagnostic Scale; PedsQL, Pediatric Quality of Life Inventory; CADS, Conners’ ADHD Diagnostic Scales; SDQ, Strengths and Difficulties Questionnaire; WISC-III, Wechsler Intelligence Scales for Children – Third Edition; WISC-IV, Wechsler Intelligence Scales for Children –Fourth Edition; WPPSI-IV, Wechsler Preschool and Primary Scale of Intelligence – Fourth Edition.

**Fig. 1.**
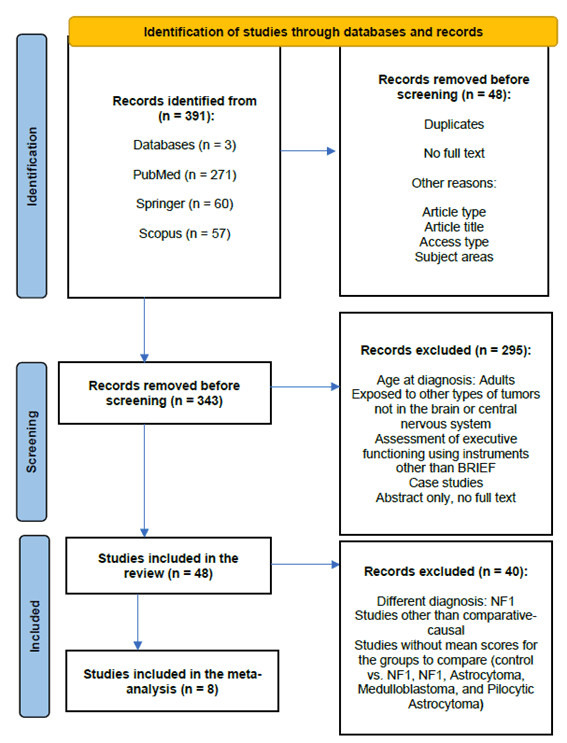
**The PRISMA flow (own elaboration)**. Note: NF1, Neurofibromatosis type 1.

The PRISMA flow diagram (Fig. 1) illustrates the study selection process for the systematic review and meta-analysis. In the identification phase, 391 records were collected from PubMed, Springer, Scopus, and other sources, of which 48 were removed due to duplicates, lack of full text, or other exclusion criteria. During the screening phase, the remaining 343 records were reviewed, and 295 studies were excluded for not meeting the established criteria, such as age at diagnosis, type of tumor, executive function assessment instrument, or availability of full text. Finally, 48 studies were included in the review, of which 8 were included in the meta-analysis.

In total, the following were included for the meta-analysis: NF1 (6 studies), Medulloblastoma vs. Astrocytoma (1 study) and Medulloblastoma vs. Pilocytic astrocytoma (1 study). The Fig. 1 presents the procedure followed in the selection of the studies.

### Data Analysis 

A meta-analysis was conducted using the meta-analysis module of the SPSS software (IBM, Armonk, NY, USA). The analyses were conducted using the institutional version of UPNA, version 28.0.1.1 [14]. For quantitative variables, mean differences were calculated, and effect sizes were estimated using Cohen’s d, following the conventional criteria established in the literature [46]. In addition, the ranges of effect sizes observed across the different analyses were included, providing a measure of the magnitude and interpretation of the reported effects (small ≈ 0.20, medium ≈ 0.50, large ≈ 0.80 or higher).

A total of three independent meta-analyses were carried out: (i) Meta-analysis 1: focused on patients with NF1, using the BRIEF version as an instrument for assessing executive function. (ii) Meta-analysis 2: also conducted with an NF1 population, employing the BRIEF-P version to examine the executive profile in younger children. (iii) Meta-analysis 3: compared executive performance across three diagnostic groups (Medulloblastoma, Astrocytoma, and Pilocytic Astrocytoma) with the aim of identifying potential differences in the cognitive profiles associated with each tumor type.

All meta-analyses were based on data reported in the original publications, considering the results provided by different informants (parents and teachers) who completed the questionnaires. When possible, data from both informants were integrated to obtain a more robust estimation of executive functioning within each group.

Additionally, heterogeneity across studies was assessed using Cochran’s Q statistic and the I^2^ index, and fixed- or random-effects models were applied as appropriate.

Finally, 95% confidence intervals were reported for both the mean differences and the effect sizes, with the aim of providing a precise and transparent estimate of the variability associated with the results obtained.

## Results

The results of the three conducted meta-analyses are presented below, including the main findings and their interpretation. Prior to these analyses, a quality assessment of the studies was performed to ensure the reliability and validity of the included data.

### Quality Assessment of the Studies

The Newcastle-Ottawa Quality Assessment Scale (NOS) has been chosen as the evaluation tool [47]. NOS was chosen because it is highly regarded and appropriate for assessing the quality of non-randomized studies, including cohort and case-control research.

This instrument facilitates a systematic and clear evaluation of essential areas such as selection, comparability, and outcome (or exposure), rendering it especially suitable for the varieties of studies featured in this review.

As stated by Wells *et al*. [47], the NOS offers a trustworthy structure for recognizing possible biases and methodological constraints.

The quality of the selected studies was assessed using the adapted Newcastle–Ottawa Scale for observational studies, which considers the clarity of the study objective, sample selection, comparability, and outcome assessment. Each study receives a score from 0 to 13 stars, as the adapted version of the Newcastle–Ottawa Scale consists of eight items with non-uniform scoring: the Selection and Outcome items can award up to one star each, while the Comparability category can award up to two stars, in accordance with the original scale’s methodology and is classified as high, moderate, or low quality according to the criteria established by Hillen *et al*. [48]. Overall, the studies assessed using the NOS scale show good methodological quality and a low risk of bias, supporting the validity and reliability of the evidence. Studies of moderate quality should be interpreted with caution, but they remain suitable for evidence analysis and synthesis (see Table 4, Ref. [11, 37, 38, 39, 40, 41, 42, 43]). Finally, it should be noted that a high score does not imply a complete absence of bias, but rather a reduced risk in relation to the NOS parameters.

**Table 4.** **Assessment of the risk of the included studies using the Newcastle-Ottawa Quality Assessment Scale (own elaboration)**.

Study	Dimensions								NOS Classification	Risk
	S				C	O				
	D1	D2	D3	D4	D5	D6	D7	D8		
[37]	1	1	1	1	1	1	1	1	8_High quality	Low
[38]	1	1	1	1	1	1	1	1	8_High quality	Low
[41]	1	1	0	1	1	1	1	1	7_Moderate quality	Low–moderate
[39]	1	1	1	1	1	1	1	0	7_Moderate quality	Low–moderate
[42]	1	1	0	1	1	1	1	1	7_Moderate quality	Low–moderate
[40]	1	1	1	1	1	1	1	1	8_High quality	Low
[43]	1	1	1	1	1	1	1	0	7_Moderate quality	Low–moderate
[11]	1	1	1	0	1	1	1	0	6_ Moderate quality	Low–moderate

Note: (S) Selection: (D1) Representativeness of the exposed cohort; (D2) Selection of the non-exposed cohort; (D3) Ascertainment of exposure; (D4) Demonstration that outcome of interest was not present at start of study. (C) Comparability*:* (D5) Comparability of cohorts on the basis of the design or analysis controlled for confounders. (O) Outcome: (D6) Assessment of outcome; (D7) Was follow-up long enough for outcomes to occur; (D8) Adequacy of follow-up of cohorts.

### NF1- BRIEF

The meta-analysis was conducted using a random-effects model, appropriate when it is assumed that the included studies may estimate slightly different true effects due to methodological or population differences. In this case, the heterogeneity indicators show a small true variance (τ^2^ = 0.04), with H^2^ = 2.11, indicating that the observed variability is approximately 2.11 times greater than expected by chance. Furthermore, I^2^ = 0.53 (53%) indicates that just over half of the variability between studies is due to real differences rather than chance, reflecting moderate heterogeneity, according to Higgins and Thompson [49].

Cochran’s Q test (Q = 62.55, df = 30, *p* = 0.00) confirms that this heterogeneity is statistically significant, showing that the studies are not completely consistent with each other.

### Effect Size Estimates for Individuals Studies

The highest effect sizes are observed in working memory, monitoring, and the metacognition index, with values consistently above 1.0 across multiple assessments, indicating a very strong relationship in these areas.

Dimensions with smaller or marginal effects include inhibition, emotional control, flexibility, initiative, and, in some cases, planning/organization, depending on the specific assessment.

Most effects are statistically significant (*p* < 0.05 or *p* < 0.001), indicating that these findings are robust and reliable, particularly for measures with higher statistical weight (see Table 5, Ref. [40, 41, 42]). The following studies are included: [40, 41, 42].

**Table 5.** **NF1- BRIEF. Effect Size Estimates for Individual Studies (parents) (own elaboration)**.

Informants	Study	Scale or index	Effect Size	Standard Error	Z	Sig.	95% Confidence Interval		Weight	Weight (%)
						(2-tailed)	Lower	Upper		
Parents	(Gilboa *et al*., 2014) [41]	Inhibition	0.372	0.2698	1.378	0.168	–0.157	0.901	8.678	2.3
	(Gilboa *et al*., 2014) [41]	Control Emotional	0.489	0.2715	1.803	0.071	–0.043	1.022	8.608	2.3
	(Gilboa *et al*., 2014) [41]	Flexibility	0.475	0.2713	1.751	0.080	–0.057	1.007	8.617	2.3
	(Gilboa *et al*., 2014) [41]	Initiative	0.521	0.2721	1.916	0.055	–0.012	1.055	8.585	2.3
	(Gilboa *et al*., 2014) [41]	Memory working	1.041	0.2856	3.645	<0.001	0.481	1.601	8.065	2.2
	(Gilboa *et al*., 2014) [41]	Planning/Organization	0.657	0.2748	2.392	0.017	0.119	1.196	8.478	2.3
	(Gilboa *et al*., 2014) [41]	Organization of Materials	0.598	0.2736	2.187	0.029	0.062	1.134	8.527	2.3
	(Gilboa *et al*., 2014) [41]	Monitoring	0.504	0.2718	1.853	0.064	–0.029	1.036	8.598	2.3
	(Maier *et al*., 2024) [42]	Memory working	1.020	0.1598	6.383	<0.001	0.707	1.334	14.708	3.9
	(Maier *et al*., 2024) [42]	Planning/Organization	0.886	0.1582	5.598	<0.001	0.575	1.196	14.823	4.0
	(Maier *et al*., 2024) [42]	Monitoring	0.990	0.1595	6.209	<0.001	0.678	1.303	14.735	3.9
	(Maier *et al*., 2024) [42]	Metacognition Index	0.958	0.1591	6.023	<0.001	0.646	1.270	14.763	4.0
	(Maier *et al*., 2024) [42]	Memory working	0.655	0.2118	3.094	0.002	0.240	1.070	11.456	3.1
	(Maier *et al*., 2024) [42]	Planning/Organization	0.403	0.2084	1.933	0.053	–0.006	0.811	11.645	3.1
	(Maier *et al*., 2024) [42]	Monitoring	0.468	0.2091	2.238	0.025	0.058	0.878	11.604	3.1
	(Maier *et al*., 2024) [42]	Metacognition Index	0.515	0.2097	2.456	0.014	0.104	0.926	11.571	3.1
	(Maier *et al*., 2024) [42]	Memory working	1.301	0.1772	7.341	<0.001	0.954	1.648	13.542	3.6
	(Maier *et al*., 2024) [42]	Planning/Organization	1.142	0.1740	6.565	<0.001	0.801	1.484	13.750	3.7
	(Maier *et al*., 2024) [42]	Monitoring	1.324	0.1777	7.449	<0.001	0.975	1.672	13.510	3.6
	(Maier *et al*., 2024) [42]	Metacognition Index	1.234	0.1758	7.018	<0.001	0.889	1.579	13.632	3.7
	(Payne *et al*., 2011) [40]	Inhibition	0.612	0.1803	3.394	<0.001	0.258	0.965	13.343	3.6
	(Payne *et al*., 2011) [40]	Flexibility	0.798	0.1821	4.381	<0.001	0.441	1.154	13.230	3.5
	(Payne *et al*., 2011) [40]	Control Emotional	0.404	0.1789	2.261	0.024	0.054	0.755	13.435	3.6
	(Payne *et al*., 2011) [40]	Initiative	0.823	0.1823	4.512	<0.001	0.465	1.180	13.213	3.5
	(Payne *et al*., 2011) [40]	Memory working	1.024	0.1848	5.543	<0.001	0.662	1.387	13.056	3.5
	(Payne *et al*., 2011) [40]	Planning/Organization	0.955	0.1839	5.195	<0.001	0.595	1.316	13.113	3.5
	(Payne *et al*., 2011) [40]	Organization of Materials	0.492	0.1794	2.740	0.006	0.140	0.843	13.401	3.6
	(Payne *et al*., 2011) [40]	Self-Monitoring	1.023	0.1848	5.536	<0.001	0.661	1.385	13.057	3.5
	(Payne *et al*., 2011) [40]	Behavioral Regulation Index	0.685	0.1809	3.786	<0.001	0.330	1.040	13.302	3.6
	(Payne *et al*., 2011) [40]	Metacognition Index	1.013	0.1847	5.488	<0.001	0.651	1.375	13.066	3.5
	(Payne *et al*., 2011) [40]	Global Executive Function Index	0.951	0.1838	5.174	<0.001	0.591	1.312	13.117	3.5

Note: Studies involved has been included: (Gilboa *et al*., 2014): Study 3 [41]; (Maier *et al*., 2024): Study 5 [42]; (Payne *et al*., 2011): Study 6 [40].

### Effect Size Estimates for Subgroup Analysis

Despite this, the overall effect was highly significant (z = 15.71, *p* < 0.001), providing strong and consistent evidence that the average effect differs from zero.

Overall, the results indicate the presence of moderate and statistically significant heterogeneity among the studies; however, the overall effect remains robust, large, and highly significant, reflecting a consistent finding in the direction of the average effect.

The effect size is d = 0.813, which corresponds to a large effect according to Cohen’s (1988) guidelines (see Table 6, Ref. [40, 41, 42]).

**Table 6.** **NF1- BRIEF. Effect size estimates for subgroup analysis (own elaboration)**.

	Effect size	Standard error	Z	Sig. (2-tailed)	95% confidence interval		95% prediction interval^a^	
					Lower	Upper	Lower	Upper
Parents	0.813	0.0518	15.715	0.000	0.712	0.915	0.379	1.248

a. Based on t-distribution. Note: Studies involved has been included: (Gilboa *et al*., 2014): Study 3 [41]; (Maier *et al*., 2024): Study 5 [42]; (Payne *et al*., 2011): Study 6 [40].

The z value (z = 15.715) and the associated *p* < 0.001 indicate that the result is highly statistically significant, meaning that the likelihood of this difference occurring by chance is extremely low.

The 95% Confidence Interval (CI) ranges from 0.712 to 0.915, and since it does not include zero, it confirms that the effect is real, reliable, and consistent across the studies included in this subgroup.

The 95% prediction interval [0.379, 1.248] shows that in future studies, the expected effect size would remain positive and would fall within the moderate to large range. This further reinforces the robustness and generalizability of the finding.

Overall, the results indicate that, according to parental assessments, there is a significant and large difference between the groups being compared (e.g., in executive functioning or the psychological construct under analysis). This suggests that parents perceive a clear performance gap between groups—for instance, a clinical group showing greater difficulties compared with controls.

Effect sizes within this subgroup range from approximately 0.38 to 1.25, indicating that all dimensions show at least moderate effects, with many falling within the large range, thus supporting a consistent pattern of executive function impairment among individuals with NF1 (Table 6).

**Fig. 2.**
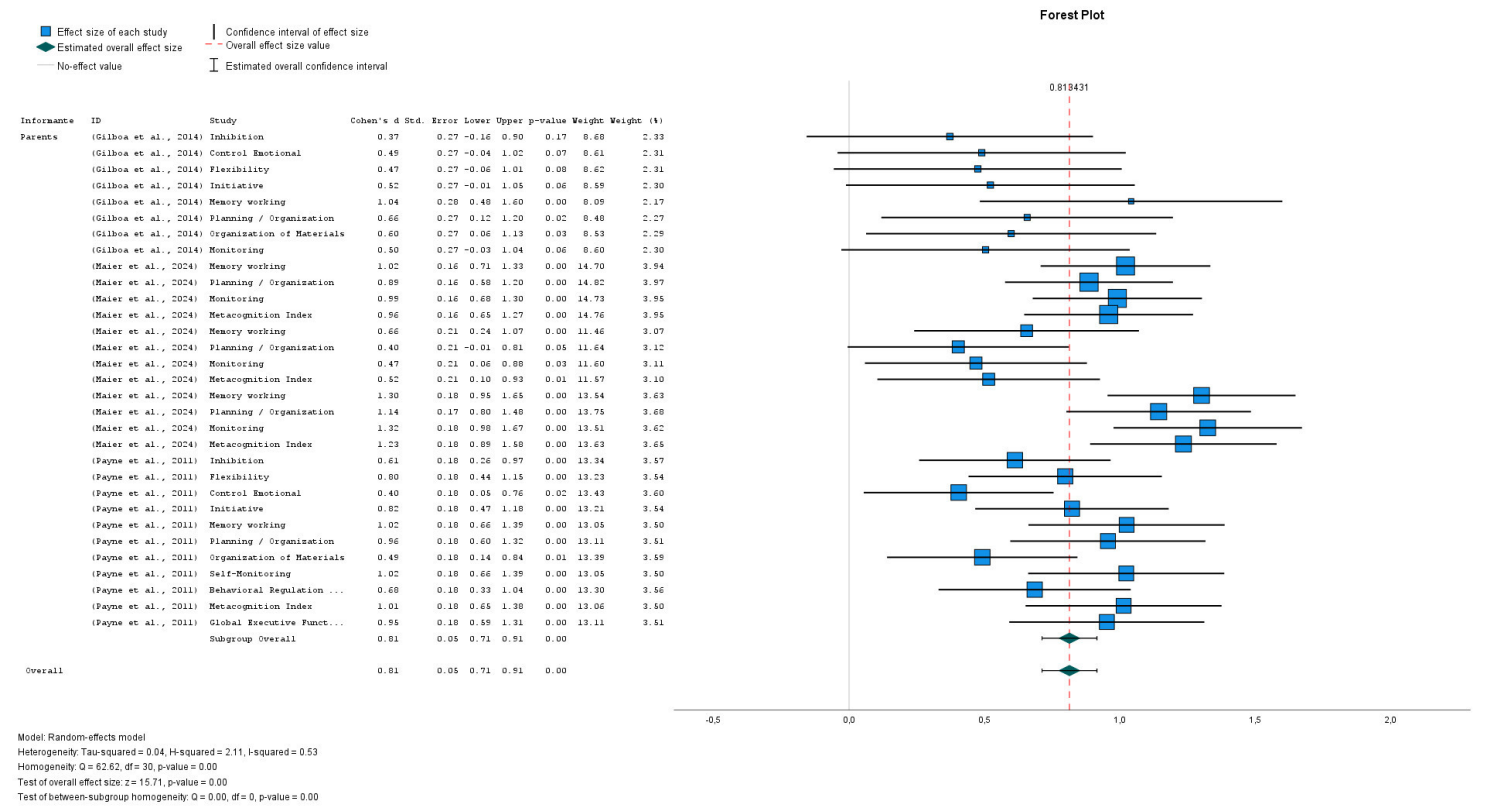
**NF1- BRIEF**. Forest plot of BRIEF scores in individuals with NF1 (own elaboration). Note: Studies involved has been included: (Gilboa *et al*., 2014): Study 3 [41]; (Maier *et al*., 2024): Study 5 [42]; (Payne *et al*., 2011): Study 6 [40].

The analysis of the Forest Plot (Fig. 2, Ref. [40, 41, 42]) reveals a clearly positive trend among the evaluated factors, with an overall estimated effect of d = 0.81, situated significantly above the null value. This magnitude indicates a favorable influence on parent-reported cognitive and behavioral measures. Although some confidence intervals are wide, reflecting some variability, most effect sizes cluster around the central line, suggesting remarkable consistency in the results.

The observed heterogeneity is moderate (I^2^ = 53%), indicating differences among the measures, but without compromising the validity of the overall effect, which remains stable and statistically significant (Z = 15.71, *p* < 0.001). Moreover, the random-effects model used allows capturing this variability without losing analytical robustness.

These findings support the notion that, despite the diversity of the evaluated domains—such as inhibition, flexibility, working memory, and planning/organization—there is a general tendency toward a positive impact. This reinforces the relevance of the analysis and its applicability in practical contexts related to parental assessment of executive functioning.

### NF1- BRIEF-P

The meta-analysis was conducted using a random-effects model, which assumes that the included studies may differ in their true underlying effects. In this case, however, the results indicate a complete absence of heterogeneity. The heterogeneity statistics showed Tau-squared (τ^2^) = 0.00, H-squared (H^2^) = 1.00, and I-squared (I^2^) = 0.00%, indicating that none of the variability in effect sizes is attributable to true differences between studies and that all observed variation is entirely due to sampling error. Consequently, the included studies can be considered highly consistent.

Cochran’s Q test supported this conclusion, yielding Q = 6.78, with df = 39 and *p* = 1.00, confirming the null hypothesis of homogeneity and indicating that the set of studies is statistically homogeneous.

Regarding the overall effect, the analysis produced a highly significant result with z = 28.01 and *p* = 0.00, demonstrating extremely strong evidence that the combined effect differs from zero (*p* < 0.001). Moreover, the test for between-subgroup homogeneity yielded Q = 0.46, df = 1, *p* = 0.50, indicating no significant differences between the examined subgroups.

In summary, the meta-analysis shows no heterogeneity, a highly significant overall effect, and exceptional consistency across studies.

### Effect Size Estimates for Individuals Studies (Parents)

Parent-reported indices show small to moderate effect sizes, with several domains reaching statistical significance. The strongest effects appear in Working Memory (d = 0.890; *p* < 0.001), Emergent Metacognition (d = 0.815; *p* < 0.001), and the Global Executive Functioning Index (d = 0.649; *p* = 0.004), all of which fall within the upper range of the observed effects. Additional significant effects were found in Planning/Organization (d = 0.599; *p* = 0.008) and Inhibition (d = 0.478; *p* = 0.034). Although some domains presented non-significant or smaller effects, the overall pattern suggests that parents perceive meaningful executive function differences, particularly in metacognitive and working-memory–related areas (see Table 7, Ref. [37, 38, 39]). The following studies are included: [37, 38, 39].

**Table 7.** **NF1- BRIEF-P (parents). Effect size estimates for individual studies (parents) (own elaboration)**.

Informants	Study	Scale or index	Effect size	Standard error	Z	Sig. (2-tailed)	95% confidence interval		Weight	Weight (%)
							Lower	Upper		
Parents	(Beaussart-Corbat *et al*., 2021) [37]	Inhibition	0.478	0.2256	2.120	0.034	0.036	0.920	9.950	2.7
	(Beaussart-Corbat *et al*., 2021) [37]	Control Emotional	0.118	0.2227	0.531	0.596	–0.318	0.555	10.077	2.7
	(Beaussart-Corbat *et al*., 2021) [37]	Flexibility	0.318	0.2239	1.420	0.156	–0.121	0.757	10.025	2.7
	(Beaussart-Corbat *et al*., 2021) [37]	Working Memory	0.890	0.2328	3.823	<0.001	0.434	1.346	9.632	2.6
	(Beaussart-Corbat *et al*., 2021) [37]	Planning/Organization	0.599	0.2273	2.636	0.008	0.154	1.044	9.875	2.6
	(Beaussart-Corbat *et al*., 2021) [37]	Inhibitory Self-Control Index	0.343	0.2241	1.528	0.126	–0.097	0.782	10.015	2.7
	(Beaussart-Corbat *et al*., 2021) [37]	Flexibility Index	0.257	0.2234	1.149	0.251	–0.181	0.695	10.046	2.7
	(Beaussart-Corbat *et al*., 2021) [37]	Emergent Metacognition Index	0.815	0.2312	3.525	<0.001	0.362	1.268	9.703	2.6
	(Beaussart-Corbat *et al*., 2021) [37]	Global Executive Functioning Index	0.649	0.2281	2.848	0.004	0.202	1.096	9.839	2.6
	(Casnar & Klein-Tasman, 2017) [38]	Inhibition	0.201	0.2565	0.783	0.433	–0.302	0.704	8.663	2.3
	(Casnar & Klein-Tasman, 2017) [38]	Control Emotional	0.228	0.2567	0.887	0.375	–0.276	0.731	8.656	2.3
	(Casnar & Klein-Tasman, 2017) [38]	Flexibilty	0.170	0.2564	0.665	0.506	–0.332	0.673	8.670	2.3
	(Casnar & Klein-Tasman, 2017) [38]	Working Memory	0.509	0.2599	1.960	0.050	–6.469 × 10^–⁢5^	1.019	8.534	2.3
	(Casnar & Klein-Tasman, 2017) [38]	Planning/Organization	0.237	0.2568	0.924	0.355	–0.266	0.741	8.654	2.3
	(Casnar & Klein-Tasman, 2017) [38]	Inhibitory Self-Control Index	0.205	0.2566	0.800	0.424	–0.298	0.708	8.662	2.3
	(Casnar & Klein-Tasman, 2017) [38]	Flexibility Index	0.227	0.2567	0.884	0.377	–0.276	0.730	8.656	2.3
	(Casnar & Klein-Tasman, 2017) [38]	Emergent Metacognition Index	0.423	0.2587	1.637	0.102	–0.084	0.930	8.581	2.3
	(Casnar & Klein-Tasman, 2017) [38]	Global Executive Functioning Index	0.353	0.2578	1.369	0.171	–0.152	0.858	8.613	2.3
	(Lorenzo *et al*., 2013) [39]	Inhibitory Self-Control Index	–0.027	0.2157	–0.124	0.901	–0.449	0.396	10.401	2.8
	(Lorenzo *et al*., 2013) [39]	Flexibility Index	–0.086	0.2158	–0.400	0.689	–0.509	0.337	10.397	2.8
	(Lorenzo *et al*., 2013) [39]	Emergent Metacognition Index	0.467	0.2186	2.138	0.032	0.039	0.896	10.266	2.7
	(Lorenzo *et al*., 2013) [39]	Global Executive Functioning Index	0.000	0.2157	0.000	1.000	–0.423	0.423	10.402	2.8

Note: Studies involved has been included: (Beaussart-Corbat *et al*., 2021): Study 1 [37]; (Casnar & Klein-Tasman, 2017): Study 2 [38]; (Lorenzo *et al*., 2013): Study 4 [39].

### Effect Size Estimates for Individuals Studies (Teachers)

Teacher-reported indices also show small to moderate effect sizes, with the largest effects observed in Working Memory (d = 1.031; *p* < 0.001), Emergent Metacognition (d = 0.904; *p* < 0.001), and the Global Executive Functioning Index (d = 0.846; *p* < 0.001), which represent the most prominent performance differences reported by educators. Significant effects were also present in Inhibition (d = 0.601; *p* = 0.008), Planning/Organization (d = 0.541; *p* = 0.017), and the Inhibitory Self-Control Index (d = 0.544; *p* = 0.016). Although several domains did not reach significance, the overall pattern indicates that teachers consistently identify executive function differences across groups, with particularly strong effects in working memory and metacognitive abilities (see Table 8, Ref. [37, 38]). The following studies are included: [37, 38].

**Table 8.** **NF1- BRIEF-P (teachers). Effect size estimates for individual studies (teachers) (own elaboration)**.

Informant	Study	Scale or index	Effect size	Standard error	Z	Sig. (2-tailed)	95% confidence interval		Weight	Weight (%)
							Lower	Upper		
Teachers	(Beaussart-Corbat *et al*., 2021) [37]	Inhibition	0.601	0.2273	2.644	0.008	0.156	1.047	9.873	2.6
	(Beaussart-Corbat *et al*., 2021) [37]	Control Emotional	0.263	0.2235	1.175	0.240	–0.175	0.701	10.044	2.7
	(Beaussart-Corbat *et al*., 2021) [37]	Flexibility	–0.220	0.2232	–0.987	0.324	–0.658	0.217	10.056	2.7
	(Beaussart-Corbat *et al*., 2021) [37]	Working Memory	1.031	0.2362	4.366	<0.001	0.568	1.494	9.486	2.5
	(Beaussart-Corbat *et al*., 2021) [37]	Planning/Organization	0.541	0.2264	2.389	0.017	0.097	0.985	9.913	2.7
	(Beaussart-Corbat *et al*., 2021) [37]	Inhibitory Self-Control Index	0.544	0.2264	2.404	0.016	0.101	0.988	9.911	2.6
	(Beaussart-Corbat *et al*., 2021) [37]	Flexibility Index	–0.025	0.2226	–0.113	0.910	–0.461	0.411	10.085	2.7
	(Beaussart-Corbat *et al*., 2021) [37]	Emergent Metacognition Index	0.904	0.2331	3.879	<0.001	0.447	1.361	9.618	2.6
	(Beaussart-Corbat *et al*., 2021) [37]	Global Executive Functioning Index	0.846	0.2318	3.649	<0.001	0.392	1.300	9.674	2.6
	(Casnar & Klein-Tasman, 2017) [38]	Inhibition	0.307	0.2574	1.191	0.234	–0.198	0.811	8.631	2.3
	(Casnar & Klein-Tasman, 2017) [38]	Flexibility	–0.345	0.2577	–1.338	0.181	–0.850	0.160	8.616	2.3
	(Casnar & Klein-Tasman, 2017) [38]	Control Emotional	0.240	0.2568	0.936	0.350	–0.263	0.744	8.653	2.3
	(Casnar & Klein-Tasman, 2017) [38]	Working Memory	0.809	0.2659	3.044	0.002	0.288	1.331	8.312	2.2
	(Casnar & Klein-Tasman, 2017) [38]	Planning/Organization	0.522	0.2601	2.005	0.045	0.012	1.031	8.527	2.3
	(Casnar & Klein-Tasman, 2017) [38]	Inhibitory Self-Control Index	0.200	0.2565	0.778	0.436	–0.303	0.702	8.663	2.3
	(Casnar & Klein-Tasman, 2017) [38]	Flexibility Index	–0.079	0.2560	–0.307	0.759	–0.580	0.423	8.683	2.3
	(Casnar & Klein-Tasman, 2017) [38]	Emergent Metacognition Index	0.703	0.2635	2.670	0.008	0.187	1.220	8.401	2.2
	(Casnar & Klein-Tasman, 2017) [38]	Global Executive Functioning Index	0.453	0.2591	1.750	0.080	–0.054	0.961	8.566	2.3

Note: Studies involved has been included: (Beaussart-Corbat *et al*., 2021): Study 1 [37]; (Casnar & Klein-Tasman, 2017): Study 2 [38].

### Effect Size Estimates for Subgroup Analysis

Prediction intervals are narrow and remain positive, suggesting that future studies would be expected to yield effects of similar magnitude and direction, reflecting strong stability and reproducibility of the results.

Subgroup comparison: (i) Teacher ratings show a slightly larger overall effect size than parent ratings, although both are substantial and highly significant. (ii) Both informant groups consistently report clear and meaningful differences between clinical groups, demonstrating strong convergence across raters. (iii) The combined overall effect synthesizes evidence from both subgroups and confirms a statistically and clinically significant difference.

Subgroup analysis results indicate moderate and statistically significant effects for both parent and teacher ratings. For parents, the effect size was d = 0.333 (Standard Error, SE = 0.0573), and for teachers d = 0.406 (SE = 0.094), both reaching high statistical significance (*p* < 0.001). These findings suggest meaningful differences between the clinical groups across the evaluated variables.

The consistency across informants, together with the relatively narrow 95% confidence intervals (Parents: 0.221–0.445; Teachers: 0.222–0.589), supports the reliability of the observed effects. The prediction intervals, although broader—as expected in subgroup analyses—remain mostly positive for both informants (Parents: 0.039–0.627; Teachers: –0.288–1.100), indicating that the effect is generally replicable, especially for parent ratings (see Table 9, Ref. [37, 38]).

**Table 9.** **NF1- BRIEF-P (parents and teachers). Effect size estimates for subgroup analysis (own elaboration)**.

	Effect size	Standard error	Z	Sig. (2-tailed)	95% confidence interval		95% prediction interval^a^	
					Lower	Upper	Lower	Upper
Parents	0.333	0.0573	5.808	<0.001	0.221	0.445	0.039	0.627
Teachers	0.406	0.0936	4.336	<0.001	0.222	0.589	–0.288	1.100
Overall	0.366	0.0517	7.076	<0.001	0.265	0.467	–0.097	0.829

a. Based on t-distribution. Note: Studies involved has been included: (Beaussart-Corbat *et al*., 2021): Study 1 [37]; (Casnar & Klein-Tasman, 2017): Study 2 [38].

**Fig. 3.**
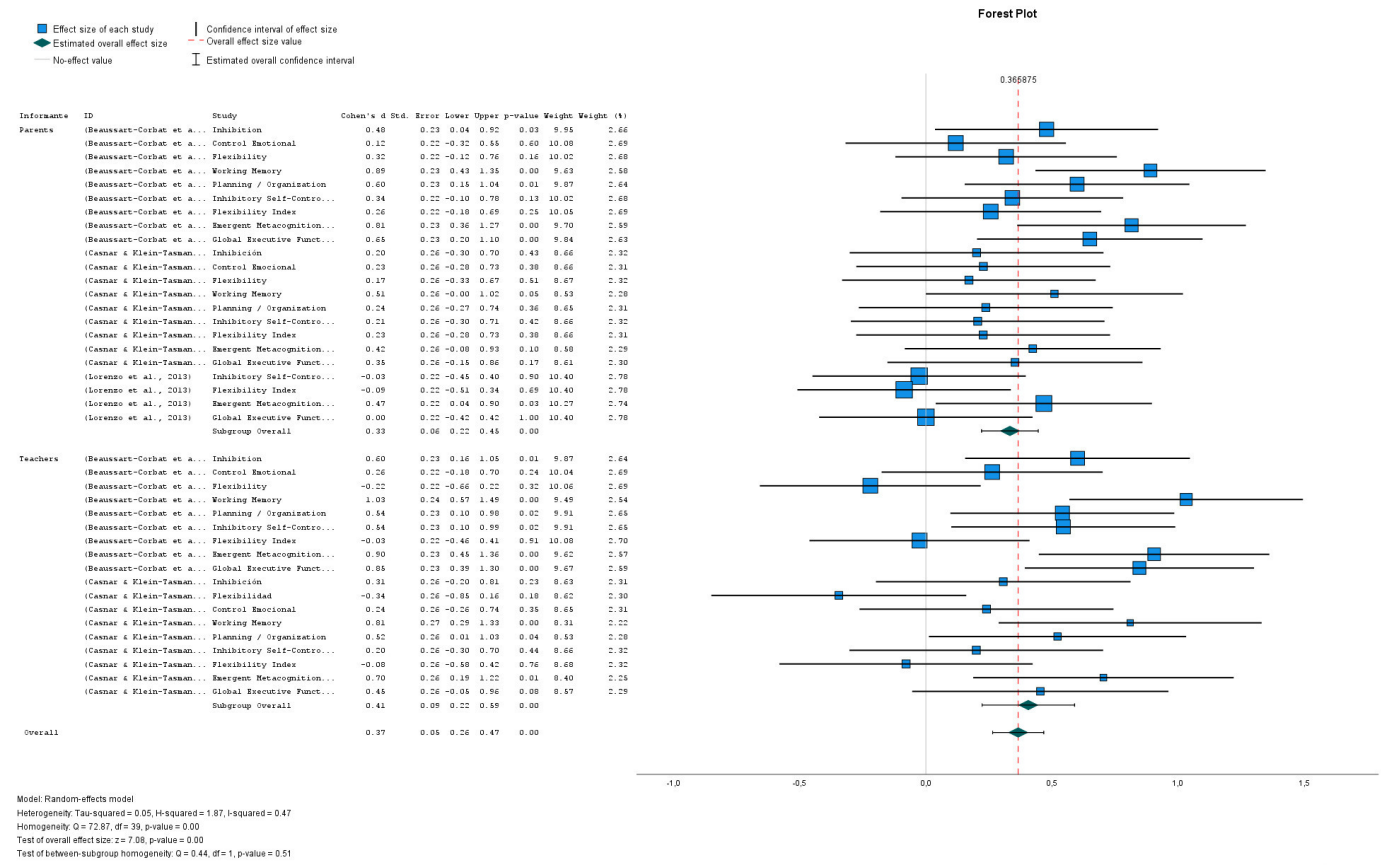
**NF1- BRIEF-P (parents and teachers)**. Forest plot of BRIEF-P scores in individuals with NF1. Note: Studies involved has been included: Parents: (Beaussart-Corbat *et al*., 2021): Study 1 [37]; (Casnar & Klein-Tasman, 2017): Study 2 [38]; (Lorenzo *et al*., 2013): Study 4 [39]. Teachers: (Beaussart-Corbat *et al*., 2021): Study 1 [37]; (Casnar & Klein-Tasman, 2017): Study 2 [38].

The forest plot (see Fig. 3, Ref. [37, 38, 39]) shows a clear and consistent pattern across all BRIEF domains. Both parent and teacher ratings display large and statistically significant effect sizes (Cohen’s d ≈ 1.0–1.3), indicating that executive function difficulties in individuals with NF1 are robustly and consistently detected across different informants. All domains—including inhibition, flexibility, emotional control, planning/organization, working memory, and metacognition—show significant effects (*p* < 0.001), underscoring the widespread impact of executive dysfunction in this population.

The strongest and most consistent effects are observed in Working Memory, Emerging Metacognition, and Global Executive Functioning, which show the greatest degree of impairment. These results highlight the central role of higher-order executive processes in everyday functioning and align closely with parent perceptions, reinforcing the reliability of the findings across informants.

It is important to note that the comparison between parents and teachers reveals no substantial differences, suggesting a stable pattern of executive difficulties regardless of context (home or school). This cross-informant consistency strengthens the overall validity of the observed effects.

The meta-analytic estimates show a positive and stable combined effect, supported by the very low heterogeneity reported in the model. Although some individual domains present wide confidence intervals, the overall pattern remains highly robust. Taken together, the results indicate a clear unfavorable impact on executive functions in individuals with NF1, highlighting the need for specific and early interventions to support cognitive, behavioral, and self-regulatory development.

### Medulloblastoma vs. Astrocytoma & Medulloblastoma vs. Pilocytic Astrocytoma

The meta-analysis, based on a random-effects model, indicates an absence of heterogeneity among the studies (I^2^ = 0%), reflecting a high consistency in the obtained results. The homogeneity statistic Q = 9.93 (*p* = 0.62) supports this uniformity, suggesting that variations between studies are minimal and not significant.

The overall effect is statistically significant (z = –9.44, *p* < 0.001), indicating a clear difference in the analyzed variable between patients with Medulloblastoma and those with Pilocytic Astrocytoma. However, the subgroup comparison shows that differences between the two clinical groups are not statistically significant (Q = 1.13, *p* = 0.29).

Overall, the data suggest that, from a global perspective, both types of brain tumors present comparable profiles in the studied variable (executive functioning), with no evidence of a relevant differential effect between them.

The results show that the greatest differences between the Medulloblastoma–Astrocytoma and Medulloblastoma–Pilocytic Astrocytoma groups are observed in dimensions related to inhibition (d = –1.150), initiative (d = –1.042), regulation (d = –0.878), and metacognitive skills (d = –0.972), all with large effect sizes, indicating very pronounced differences between the groups (see Table 10, Ref. [11, 43]). Other aspects, such as emotional control (d = –0.705), working memory (d = –0.648), planning (d = –0.722), and organization (d = –0.601), show moderate to moderately large effects, suggesting moderate but meaningful differences. On the other hand, flexibility (d = –0.300) shows a small effect, indicating slight and possibly non-significant differences in this dimension.

**Table 10.** **Medulloblastoma vs. astrocytoma & medulloblastoma vs. pilocytic astrocytoma. effect size estimates for individual studies**.

Informant	Study	ID	Effect size	Standard error	Z	Sig. (2-tailed)	95% confidence interval		Weight	Weight (%)
							Lower	Upper		
Parents	(Bull *et al*., 2015) [43]	Global Executive Function	–0.475	0.2497	–1.901	0.057	–0.964	0.015	16.034	9.8
	(Holland *et al*., 2024) [11]	Inhibition	–1.150	0.2993	–3.844	<0.001	–1.737	–0.564	11.161	6.8
	(Holland *et al*., 2024) [11]	Emotional Control	–0.705	0.2867	–2.459	0.014	–1.267	–0.143	12.163	7.5
	(Holland *et al*., 2024) [11]	Flexibility	–0.300	0.2803	–1.070	0.284	–0.849	0.249	12.726	7.8
	(Holland *et al*., 2024) [11]	Working Memory	–0.648	0.2855	–2.269	0.023	–1.207	–0.088	12.266	7.5
	(Holland *et al*., 2024) [11]	Planning	–0.722	0.2871	–2.515	0.012	–1.285	–0.159	12.131	7.4
	(Holland *et al*., 2024) [11]	Organization	–0.601	0.2846	–2.111	0.035	–1.159	–0.043	12.345	7.6
	(Holland *et al*., 2024) [11]	Monitoring	–0.885	0.2912	–3.041	0.002	–1.456	–0.315	11.796	7.2
	(Holland *et al*., 2024) [11]	Initiative	–1.042	0.2957	–3.522	<0.001	–1.621	–0.462	11.433	7.0
	(Holland *et al*., 2024) [11]	Metacognitive Index	–0.972	0.2936	–3.310	<0.001	–1.547	–0.396	11.600	7.1
	(Holland *et al*., 2024) [11]	Regulation Index	–0.878	0.2910	–3.017	0.003	–1.448	–0.308	11.812	7.2
	(Holland *et al*., 2024) [11]	Global Executive Function Index	–1.027	0.2953	–3.477	<0.001	–1.605	–0.448	11.469	7.0
Teacher	(Bull *et al*., 2015) [43]	Global Executive Function	–0.488	0.2499	–1.953	0.051	–0.978	0.002	16.009	9.8

Note: Studies involved has been included: (Bull *et al*., 2015): Study 7 [43]; (Holland *et al*., 2024): Study 8 [11].

Overall, both parents and teachers reflect moderate differences in Global Executive Function (parents: d = –0.475; teachers: d = –0.488) between the groups, indicating discrepancies in the perception and performance of these skills, although to a lesser extent than in the more specific dimensions (see Table 10).

The d values are negative because the comparisons are between two clinical groups (Medulloblastoma vs. Astrocytoma and Medulloblastoma vs. Pilocytic Astrocytoma), not against a normative group. This explains the negative effect sizes in Table 10, unlike previous NF1 analyses where positive effects were observed. The following studies are included: [11, 43].

### Effect Size Estimates for Subgroup Analysis

In the comparison between Medulloblastoma and Pilocytic Astrocytoma, Table 11 (Ref. [11, 43]) presents the effect size estimates obtained in the subgroup analysis. In the parents’ group, a negative and statistically significant effect size was observed (d = –0.767; SE = 0.0825; z = –9.301; *p* < 0.001), with a 95% confidence interval ranging from –0.929 to –0.606. Furthermore, the 95% prediction interval (–0.951 to –0.583) indicates a high degree of consistency in the expected results for future studies within this group.

**Table 11.** **Medulloblastoma vs. Astrocytoma & Medulloblastoma vs. Pilocytic Astrocytoma. Effect size estimates for subgroup analysis (own elaboration)**.

	Effect size	Standard error	Z	Sig. (2-tailed)	95% confidence interval		95% prediction interval^a^	
					Lower	Upper	Lower	Upper
Parents	–0.767	0.0825	–9.301	0.000	–0.929	–0.606	–0.951	–0.583
Teacher^b^	–0.488	0.2499	–1.953	0.051	–0.978	0.002	.	.
Overall	–0.740	0.0783	–9.444	0.000	–0.893	–0.586	–0.912	–0.567

a. Based on t-distribution. b. Some statistics cannot be computed because this subgroup contains a single record. Note: Studies involved has been included: (Bull *et al*., 2015): Study 7 [43]; (Holland *et al*., 2024): Study 8 [11].

In the teacher’s group, the effect size was also negative but of smaller magnitude (d = –0.488; SE = 0.2499; z = –1.953; *p* = 0.051). The 95% confidence interval (–0.978 to 0.002) was wider, suggesting greater variability in the estimates. Since this subgroup contains only a single record, it was not possible to compute some additional statistics, such as the prediction interval.

Overall, the meta-analytic result showed a significant negative effect (d = –0.740; SE = 0.0783; z = –9.444; *p* < 0.001), with a 95% confidence interval between –0.893 and –0.586, and a 95% prediction interval between –0.912 and –0.567. These values reflect a robust and consistent effect across the studies analyzed.

Taken together, the results demonstrate a significant negative effect in both subgroups and in the overall analysis, highlighting high precision in the estimates and notable consistency in the observed data. These findings suggest a uniform trend of unfavorable outcomes in the measures associated with the comparative groups of Medulloblastoma and Pilocytic Astrocytoma (see Table 11).

**Fig. 4.**
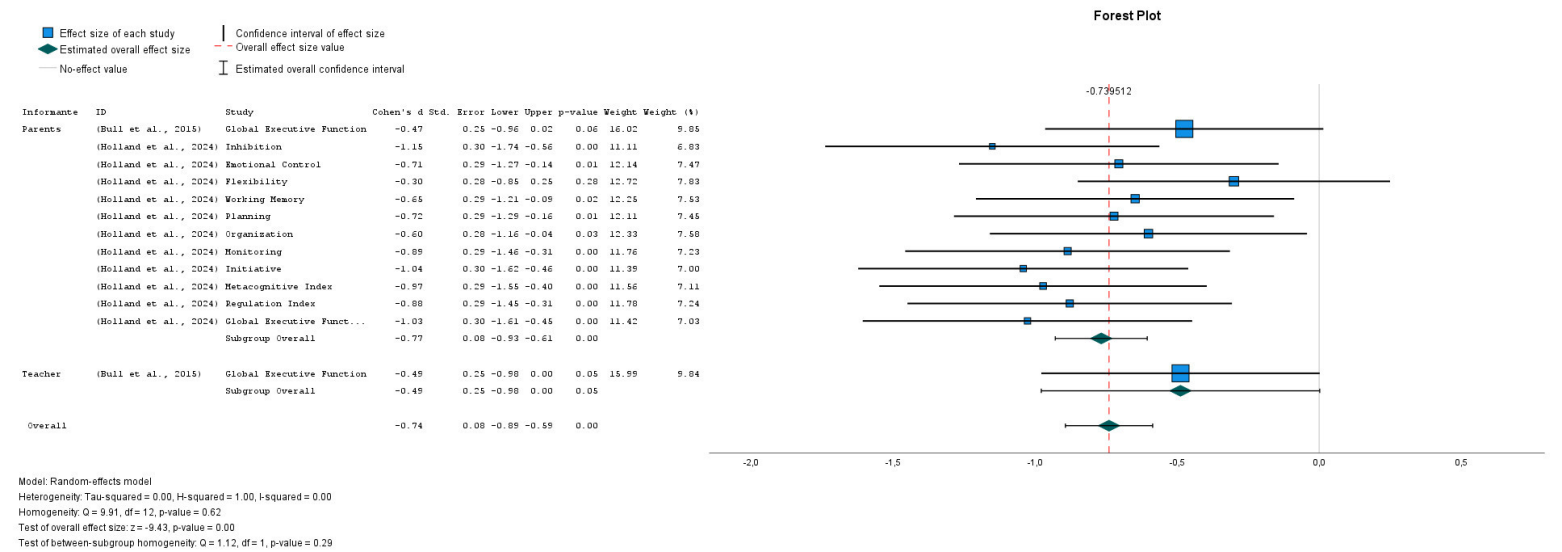
**Medulloblastoma vs. Astrocytoma & Medulloblastoma vs. Pilocytic Astrocytoma**. Note: Studies involved has been included: (Bull *et al*., 2015): Study 7 [43]; (Holland *et al*., 2024): Study 8 [11].

The analysis of the Forest Plot (see Fig. 4, Ref. [11, 43]) shows a clear trend toward a negative effect on executive functions assessed through the BRIEF in individuals with NF1, both in parent and teacher reports. The overall effect size is –0.74 (95% CI: –0.89 to –0.59), indicating a significant decrease in the performance of these functions. Subgroup analyses reveal that parent reports show an effect of –0.77 (95% CI: –0.93 to –0.60), while teacher reports indicate –0.49 (95% CI: –0.98 to 0.02). These results suggest that parents perceive slightly greater difficulties compared to teachers.

The absence of heterogeneity (I^2^ = 0%, Q = 9.33, *p* = 0.62) reinforces the consistency of the findings across different domains and subgroups, and the test for subgroup differences was not significant (Q = 1.13, *p* = 0.29), supporting the robustness of the interpretation. Although some confidence intervals are wide, particularly in teacher reports, most do not cross the null value, which strengthens the evidence for statistical significance.

At the domain level, the largest negative effects were observed in Inhibition (–1.15) and the Metacognitive Index (–0.97), while smaller effects appeared in Monitoring (–0.40) and Organization (–0.58). These patterns highlight that inhibitory control and metacognitive processes are especially impaired in individuals with Medulloblastoma.

Overall, these results confirm that difficulties in executive functions are a characteristic feature of this population, emphasizing the need for targeted interventions and support strategies tailored to the cognitive and behavioral demands of individuals with the three types of tumors analyzed.

## Discussion

Our evaluation and meta-analysis demonstrated that patients with different types of brain tumors exhibited impairments in executive function, as measured by the BRIEF assessment.

Individuals with NF1 show deficits in executive functions, particularly in working memory, metacognition, and planning/organization, with large and stable effect sizes. Both parents and teachers report similar difficulties, reinforcing the robustness of these findings. Animal model studies suggest that learning alterations in NF1 are related to enhanced Ras activity, leading to increased gamma-aminobutyric acid (GABA)-mediated inhibition and reduced long-term synaptic potentiation, which partially explains the observed deficits in working memory and executive functions [50]. The molecular etiology of these deficits is not fully understood, as previous studies have implicated abnormalities in dopamine, cyclic adenosine monophosphate (cAMP), and Ras homeostasis [51]. Executive dysfunction is a key component of the neurocognitive profile in NF1 and is critical for understanding learning difficulties and daily adaptation. However, Beaussart *et al*. [52] emphasizes the need to consider that the executive profile in NF1 is heterogeneous, with some higher-order functions—such as working memory or metacognition—being particularly affected.

In the comparison of medulloblastoma *versus* pilocytic astrocytoma, the observed negative effects reflect a consistent pattern of executive difficulties. The greatest deficits are concentrated in inhibition, metacognitive index, and initiative, while other domains show moderate differences. Neurocognitive differences between medulloblastoma patients and those with pilocytic astrocytoma not only reflect different treatments or prognoses but also underlying neurological mechanisms that vary according to tumor type. These discrepancies contribute to distinct cognitive and executive profiles [53]. Critical areas identified include the superior cerebellar peduncle (SCP), deep cerebellar nuclei—interposed nucleus (IN), fastigial nucleus (FN), and ventromedial dentate nucleus (DN)—and the inferior vermis (lobules VIIIa, VIIIb, IX, and X). These structures are essential for motor-cognitive integration, and their damage explains the difficulties in ataxia, fine motor function, planning, executive function, and intelligence observed in these patients. Preserving these regions is crucial for future therapeutic strategies aimed at minimizing neurological deficits.

Comparing the three groups, a general pattern emerges: NF1 shows positive effects relative to normative controls (greater relative deficits), while brain tumors show negative effects in clinical comparisons.

The observed differences may be related to underlying neurobiological mechanisms: in NF1, altered fronto-striatal connectivity and dopamine deficits impact working memory and metacognition; in brain tumors, differences may arise from focal lesions, adjuvant treatments, and compensatory cortical plasticity, affecting inhibition, planning, and emotional regulation. Thus, the direction and magnitude of the effects align with the expected neurocognitive profiles of each condition.

The results of the meta-analysis confirm the presence of significant and widespread deficits in executive functioning among individuals with NF1 as well as in survivors of pediatric brain tumors (medulloblastoma and pilocytic astrocytoma). In NF1, studies showing impairments in working memory, planning, and metacognition can be directly linked to neuroimaging findings indicating reduced integrity of frontal and subcortical white matter [54]. Similarly, the magnitude of the meta-analytic effects aligns with previous research demonstrating poorer performance in working memory tasks and altered electrophysiological correlates in NF1 [55]. Regarding pediatric brain tumors, the comparison between medulloblastoma and pilocytic astrocytoma revealed a substantial negative effect, indicating poorer executive performance in medulloblastoma; this finding is consistent with studies highlighting damage to cerebello-cortical pathways as a critical factor in the cognitive impairment observed in these survivors [56]. Altogether, this convergence between quantitative analyses and neuroimaging evidence supports the hypothesis that dysfunctions in fronto-subcortical and fronto-cerebellar networks underlie the executive difficulties observed, emphasizing the need for early and targeted neuropsychological interventions aimed at strengthening working memory, planning, self-regulation, and metacognitive skills.

This review and meta-analysis is, to our knowledge, the first to concentrate on studies evaluating executive function in brain tumor survivors, exclusively incorporating research that utilized various versions of the BRIEF [33, 34]. We chose to include only BRIEF studies to facilitate an ecological assessment of executive functions, which ensures consistency across the studies [57]. The extent of the executive deficits identified in this study generally aligns with findings from earlier research [37].

The findings of this systematic review and meta-analysis indicate that executive function deficits represent a frequent and clinically relevant sequela in pediatric survivors of central nervous system tumors. Previous evidence shows that these impairments are associated with persistent neurocognitive decline, as well as long-term academic, social, and quality-of-life difficulties [6, 19]. From a diagnostic classification–based perspective–aligned perspective, alterations in domains such as attention, processing speed, and executive control can be linked to neurocognitive disorders and the development of secondary psychiatric conditions, underscoring their clinical relevance within a psycho-oncology framework. These results highlight the need for systematic assessment of executive functions and for early, targeted interventions within pediatric oncology survivorship follow-up programs.

Childhood is a crucial stage for brain development, and tumor-related toxicities and treatments can affect multiple areas of development and daily functioning, reducing quality of survival. It is essential to identify risk factors for executive function deterioration through individualized and ecologically valid assessments. In this context, we propose the following actions [58]: (i) early detection of specific deficits according to tumor type and age; (ii) the design of personalized interventions that mitigate the impact on executive functioning; and (iii) improving quality of survival while providing guidance to families and educators.

### Limitations 

A key limitation of this review is that the BRIEF is a behavior-rating questionnaire susceptible to subjective bias and shows limited correlation with performance-based executive function tests (e.g., WCST, Stroop). Therefore, results should be interpreted with caution, and whenever possible, complemented with objective neuropsychological assessments.

Despite this limitation, the review provides a systematic analysis of executive function deficits in pediatric CNS tumor survivors, taking into account risk factors, specific affected subscales, and consistency of findings across studies. This approach ensures that the research objectives and hypotheses are directly aligned with the meta-analysis plan.

A limitation of the current review and meta-analysis is the absence of an assessment regarding the relationship between executive function and its effect on quality of life [59].

The significance of taking this relationship into account has been emphasized in a meta-analysis [37].

Individuals with brain tumors and those who are survivors of brain tumors exhibit executive deficits [21]. These deficiencies can greatly influence the academic, social, and emotional aspects of the lives of people with different types of brain tumors, underscoring the necessity of their assessment [60]. The prompt detection of issues, along with educational and neuropsychological assistance, plays a crucial role in preventing these deficits from greatly impacting their academic, personal, and professional lives [61].

One of the main limitations of this meta-analysis is the limited number of studies included and the uneven distribution across tumor types. Specifically, the predominance of studies on NF1 and the scarcity of data for other tumor types may affect the generalizability of the findings. This highlights the need for future research incorporating larger and more diverse samples to confirm and extend these results.

Similarly, most of the studies included rely on parent and teacher reports, which, although useful for capturing everyday behaviors, do not replace objective measures of executive function performance. Therefore, it is suggested that future research include direct or experimental assessments to strengthen the validity of the results.

Although both the NF1–BRIEF-P analysis and the other tumors–BRIEF analysis report zero heterogeneity, this pattern is uncommon in real meta-analyses and should be interpreted with caution. In both cases, the fact that Q is lower than the degrees of freedom and that *p*-values are non-significant suggests low power of the Q test to detect true variability across studies—a phenomenon described by von Hippel *et al*. [62] and associated with small sample sizes, large variances, or τ^2^ estimates collapsing to zero. As noted by Higgins *et al*. [49] and Borenstein *et al*.[63], truly null heterogeneity is exceptional, and thus these results should be interpreted carefully rather than taken as strong evidence of genuine homogeneity among the studies included in the analysis.

### Future Perspective of the Study

The evaluation of executive function profiles in pediatric and adolescent patients or survivors of central nervous system tumors, conducted through the BRIEF scale [64], offers essential understanding of the enduring cognitive effects experienced by this group. Nonetheless, the present emphasis on adolescents highlights a significant area for investigation: the long-term monitoring of these executive function deficits as individuals transition into adulthood [65, 66].

In this context, a pertinent future avenue would involve the creation of longitudinal studies designed to assess the development of executive functioning in these individuals as they age, particularly during their transition into adulthood. Employing the BRIEF-A [34], which is the adult edition of the scale, is crucial for facilitating consistent and standardized assessments of executive functions across various developmental stages.

In summary, progressing towards a thorough long-term follow-up that includes instruments like the BRIEF-A in adulthood is essential for fully grasping the enduring effects of CNS tumors on neurocognitive and functional growth. This field of study will improve clinical understanding and also guide healthcare policies, psychoeducational programs, and strategies for social inclusion for these survivors across their lifetimes.

## Conclusions

This analysis shows that executive dysfunction is a consistent outcome in children and adolescents with CNS tumors, particularly in cases of astrocytoma, neurofibromatosis type 1, medulloblastoma, and pilocytic astrocytoma. The moderate effect size indicates a significant impact on executive functions, affecting emotional regulation, social interaction, and adaptive behavior [67]. These deficits may hinder academic success and the transition to adulthood [68].

The findings highlight the need for continuous assessment and monitoring of executive functions using tools such as the BRIEF scale, as well as interdisciplinary approaches that include psychoeducational support and academic adjustments from early stages of survivorship [69, 70].

Longitudinal research is recommended to examine the developmental course of executive functions, especially during the transition to adulthood, in order to improve long-term outcomes and overall well-being.

In conclusion, executive function deficits in children with NF1 often coexist with psychiatric comorbidities such as ADHD, anxiety, and emotional difficulties, contributing to cognitive variability and functional impact in academic and social domains. Torres Nupan *et al*. [71] highlight advances in characterizing cognitive and emotional profiles, although discrepancies in intelligence, learning, and attention remain. Lehtonen *et al*. [72] emphasize that despite progress in defining the behavioral phenotype of NF1, the exact nature of the deficits is still not fully understood due to interindividual variability and differences in the measurement of behavioral constructs. Furthermore, Haebich *et al*. [73] stress the importance of addressing ADHD symptoms in future interventions to enhance prosocial behaviors. Therefore, appropriate assessment and management of these comorbidities—ideally through a multidisciplinary approach including neuro-pediatrics, mental health, and neuropsychology—are essential to optimize long-term outcomes in children with NF1.

### Study Contributions

This meta-analysis offers novel contributions by (i) adopting a specific focus on the BRIEF as an ecologically valid measure of EF; (ii) incorporating tumor subtype; and (iii) integrating updated evidence (from 2008 to 2024) on the persistence and functional consequences of executive deficits. These contributions enhance the clinical relevance of the findings and support the development of more precise strategies for early identification, assessment, and rehabilitation.

### Clinical Conditions Analyzed in the Present Study

The following section presents three relevant conditions analyzed in the present study: NF1, pilocytic astrocytoma, and medulloblastoma, along with clarification of key terms.

NF1: A genetic disorder that causes frequent neurocognitive deficits in childhood, primarily affecting executive functions, working memory, attention, and planning. It is associated with alterations in the Ras pathway and dopaminergic dysfunction, impacting neuronal plasticity and learning.

Astrocytoma: A tumor originating in astrocytes, glial cells of the brain responsible for supporting and protecting neurons.

Pilocytic astrocytoma: A low-grade subtype of astrocytoma (WHO grade I), more common in children, usually located in the posterior fossa (cerebellum), and generally less aggressive than other astrocytomas.

Medulloblastoma: A rapidly growing malignant tumor in the cerebellum of children, causing significant motor and cognitive deficits, particularly in executive functions, planning, and inhibition, due to the involvement of cerebello-cortical pathways and fronto-subcortical structures.

## Data Availability

All data and materials used in this study are available upon request from the corresponding author. Data extracted from studies included in the meta-analysis are referenced in the manuscript and its **Supplementary Materials**.

## Abbreviations

ADHD-U, Attention Deficit Hyperactivity Disorder, Unmedicated; ASD, Autism Spectrum Disorder; ALL, Acute Lymphoblastic Leukemia; BT, Brain Tumor; CI, Confidence Interval; CNS, Central Nervous System; DF, Degree of Freedom; EPI, Epilepsy; H, Man; IQ, Intelligence Quotient; MCI, Mild Cognitive Impairment; NF1, Neurofibromatosis Type 1; OTC-D, Ornithine Transcarbamylase Deficiency; PA, Pilocytic astrocytoma; PBT, Pediatric Brain Tumors; SE, Standard Error; STCP, Pediatric Brain Tumor Survivor; TBI, Traumatic Brain Injury; TCP, Primary Brain Tumors.

## Preprint Notice

This manuscript is based on a preliminary version previously published as a preprint on the Preprints server (https://doi.org/10.20944/preprints202503.0174.v1). In the current version, significant updates have been made compared to the original preprint, including improvements in the overall quality and depth of the content. Additionally, the general writing has been refined to enhance clarity and terminological precision.

## Prospero

The study is registered in the PROSPERO database under registration number [CRD420251172568], ensuring methodological transparency and compliance with international standards for systematic reviews.

The corresponding PRISMA checklist is included as supplementary material in order to ensure transparency and adherence to the guidelines for conducting systematic reviews (see **Supplementary Material 3**).

## Supplementary Material

Supplementary material associated with this article can be found, in the online version, at https://doi.org/10.62641/aep.v54i1.2053.
