# Supplementary material for: A Systematic Review and Meta-Analysis of Executive Function Outcomes in Pediatric Central Nervous System Tumor Survivors
Source: Actas Esp Psiquiatr. 2026 Feb 15;54(1):237–62. doi: 10.62641/aep.v54i1.2053 (PMC12946733; doi:10.62641/aep.v54i1.2053)
Supplement: Supplementary file 1 [file ActEsp-54-1-237-262-s1.zip › Supplementary Material 1_Search+Strategy.docx]

**Supplementary Material 1: Search Strategy**

The search strategy used to gather the literature for this review is detailed below.

**Database 1: Pubmed**

**Search strategies**

**#1# [(CANCER) OR (TUMOR)] AND [(BRIEF) OR (EXECUTIVE FUNCTION**)],,,"((""cancer s""[All Fields] OR ""cancerated""[All Fields] OR ""canceration""[All Fields] OR ""cancerization""[All Fields] OR ""cancerized""[All Fields] OR ""cancerous""[All Fields] OR ""neoplasms""[MeSH Terms] OR ""neoplasms""[All Fields] OR ""cancer""[All Fields] OR ""cancers""[All Fields] OR (""cysts""[MeSH Terms] OR ""cysts""[All Fields] OR ""cyst""[All Fields] OR ""neurofibroma""[MeSH Terms] OR ""neurofibroma""[All Fields] OR ""neurofibromas""[All Fields] OR ""tumor s""[All Fields] OR ""tumoral""[All Fields] OR ""tumorous""[All Fields] OR ""tumour""[All Fields] OR ""neoplasms""[MeSH Terms] OR ""neoplasms""[All Fields] OR ""tumor""[All Fields] OR ""tumour s""[All Fields] OR ""tumoural""[All Fields] OR ""tumourous""[All Fields] OR ""tumours""[All Fields] OR ""tumors""[All Fields])) AND (""brief""[All Fields] OR ""briefed""[All Fields] OR ""briefing""[All Fields] OR ""briefings""[All Fields] OR ""briefs""[All Fields])) OR (""executive function""[MeSH Terms] OR (""executive""[All Fields] AND ""function""[All Fields]) OR ""executive function""[All Fields])","64,465",12:23:06

**Results: 64.465 Results**

**Search strategies**

**#2# (CANCER) OR (TUMOR)] AND [(BRIEF) OR (EXECUTIVE FUNCTION) 🡪**

#2,[(CANCER) OR (TUMOR)] AND [(BRIEF) OR (EXECUTIVE FUNCTION)],,"Free full text, Full text","(((""cancer s""[All Fields] OR ""cancerated""[All Fields] OR ""canceration""[All Fields] OR ""cancerization""[All Fields] OR ""cancerized""[All Fields] OR ""cancerous""[All Fields] OR ""neoplasms""[MeSH Terms] OR ""neoplasms""[All Fields] OR ""cancer""[All Fields] OR ""cancers""[All Fields] OR (""cysts""[MeSH Terms] OR ""cysts""[All Fields] OR ""cyst""[All Fields] OR ""neurofibroma""[MeSH Terms] OR ""neurofibroma""[All Fields] OR ""neurofibromas""[All Fields] OR ""tumor s""[All Fields] OR ""tumoral""[All Fields] OR ""tumorous""[All Fields] OR ""tumour""[All Fields] OR ""neoplasms""[MeSH Terms] OR ""neoplasms""[All Fields] OR ""tumor""[All Fields] OR ""tumour s""[All Fields] OR ""tumoural""[All Fields] OR ""tumourous""[All Fields] OR ""tumours""[All Fields] OR ""tumors""[All Fields])) AND (""brief""[All Fields] OR ""briefed""[All Fields] OR ""briefing""[All Fields] OR ""briefings""[All Fields] OR ""briefs""[All Fields])) OR (""executive function""[MeSH Terms] OR (""executive""[All Fields] AND ""function""[All Fields]) OR ""executive function""[All Fields])) AND ((ffrft[Filter]) AND (fft[Filter]))","28,955",12:33:13

**Results: 67.045 results**

**Filters, limits, and justification:**

**Búsqueda 1.1:**

- Search Terms: (CANCER) OR (TUMOR) AND (BRIEF) OR (EXECUTIVE FUNCTION)

- Applied Filters: Full text, Child: 6-12 years, Preschool Child: 2-5 years, desde 2010 hasta 2024

**- Results: 6.894 Results**

**Búsqueda 1.2:**

- Search Terms: (CANCER) OR (TUMOR) AND (BRIEF) OR (EXECUTIVE FUNCTION)

#3,[(CANCER) OR (TUMOR)] AND [(BRIEF) OR (EXECUTIVE FUNCTION)],,Full text,"(((""cancer s""[All Fields] OR ""cancerated""[All Fields] OR ""canceration""[All Fields] OR ""cancerization""[All Fields] OR ""cancerized""[All Fields] OR ""cancerous""[All Fields] OR ""neoplasms""[MeSH Terms] OR ""neoplasms""[All Fields] OR ""cancer""[All Fields] OR ""cancers""[All Fields] OR (""cysts""[MeSH Terms] OR ""cysts""[All Fields] OR ""cyst""[All Fields] OR ""neurofibroma""[MeSH Terms] OR ""neurofibroma""[All Fields] OR ""neurofibromas""[All Fields] OR ""tumor s""[All Fields] OR ""tumoral""[All Fields] OR ""tumorous""[All Fields] OR ""tumour""[All Fields] OR ""neoplasms""[MeSH Terms] OR ""neoplasms""[All Fields] OR ""tumor""[All Fields] OR ""tumour s""[All Fields] OR ""tumoural""[All Fields] OR ""tumourous""[All Fields] OR ""tumours""[All Fields] OR ""tumors""[All Fields])) AND (""brief""[All Fields] OR ""briefed""[All Fields] OR ""briefing""[All Fields] OR ""briefings""[All Fields] OR ""briefs""[All Fields])) OR (""executive function""[MeSH Terms] OR (""executive""[All Fields] AND ""function""[All Fields]) OR ""executive function""[All Fields])) AND (fft[Filter])","60,228",12:33:10

- Applied Filters: Full text, Randomized Controlled Trial, Preschool Child: 2-5 years, Child: 6-12 years, desde 2010 hasta 2024

**- Results: 501 Results**

**Búsqueda 1.3:**

- Search Terms: (CANCER) OR (TUMOR) AND (BRIEF) OR (EXECUTIVE FUNCTION)

#4,[(CANCER) OR (TUMOR)] AND [(BRIEF) OR (EXECUTIVE FUNCTION)],,,"((""cancer s""[All Fields] OR ""cancerated""[All Fields] OR ""canceration""[All Fields] OR ""cancerization""[All Fields] OR ""cancerized""[All Fields] OR ""cancerous""[All Fields] OR ""neoplasms""[MeSH Terms] OR ""neoplasms""[All Fields] OR ""cancer""[All Fields] OR ""cancers""[All Fields] OR (""cysts""[MeSH Terms] OR ""cysts""[All Fields] OR ""cyst""[All Fields] OR ""neurofibroma""[MeSH Terms] OR ""neurofibroma""[All Fields] OR ""neurofibromas""[All Fields] OR ""tumor s""[All Fields] OR ""tumoral""[All Fields] OR ""tumorous""[All Fields] OR ""tumour""[All Fields] OR ""neoplasms""[MeSH Terms] OR ""neoplasms""[All Fields] OR ""tumor""[All Fields] OR ""tumour s""[All Fields] OR ""tumoural""[All Fields] OR ""tumourous""[All Fields] OR ""tumours""[All Fields] OR ""tumors""[All Fields])) AND (""brief""[All Fields] OR ""briefed""[All Fields] OR ""briefing""[All Fields] OR ""briefings""[All Fields] OR ""briefs""[All Fields])) OR (""executive function""[MeSH Terms] OR (""executive""[All Fields] AND ""function""[All Fields]) OR ""executive function""[All Fields])","64,465",12:23:06

- Applied Filters: Free full text, Full text, Randomized Controlled Trial, Preschool Child: 2-5 years, Child: 6-12 years, desde 2010 hasta 2024

**- Results: 271 Results**

**Justification of the Filters:**

The following filters and limits were applied to obtain more specific and relevant results for research studying the relationship between cancer/tumor and executive functions in children, with a particular focus on interventions and controlled studies:

**Full text:** Selected to access the complete text of the studies and review all relevant information.

**Randomized Controlled Trial:** Limits the results to studies following a randomized controlled trial design, considered one of the gold standards in clinical research for establishing the effectiveness of interventions.

**Preschool Child: 2–5 years, Child: 6–12 years:** Defines the age range of participants to focus on studies including children in specific age groups relevant to cognitive and executive development.

**From 2010–2024:** Restricts the search to studies published within the last 14 years to ensure the relevance of recent findings.

**Database 2: Springer Link**

**Search strategies 1:**

- Search Terms: [(CANCER) OR (TUMOR)] AND [(BRIEF) OR (EXECUTIVE FUNCTION)]

**Results without Filters: 10.000 Results**

**Applied Filters and Justification:**

Research article: Limits the results to academic research articles, excluding other types of documents such as reviews or commentaries, to ensure the quality and relevance of the studies.

2010–2024: Restricts the search to studies published in the last 14 years to maintain the currency of the findings.

Psychology: Includes studies specifically categorized within the field of psychology, which is relevant for research on executive functions and their relationship with cancer or tumors.

Neurology: Includes studies categorized under neurology, which is pertinent for research examining neurocognitive aspects related to executive functions in patients with cancer or tumors.

**Results after applying filters:**

Results: After applying these filters, 60 results were obtained. These selected studies should provide a solid research foundation focused on executive functions and their relationship with cancer or tumors, within the fields of psychology and neurology.

These search strategies and applied filters ensure that the results are relevant, high-quality, and pertinent to specific research on executive functions in patients with cancer or tumors, contributing to the advancement of knowledge in this field.

**Results after applying filters: 60 results**

**Database 3: SCOPUS**

**Search Strategie 3:**

**Filter 3.1: Psicología**

- Search Terms: ([cancer OR tumor]) AND [(brief OR (executive AND function))]

- Applied Filters:

- LIMIT-TO (SUBJAREA, "PSYC"): Limita la búsqueda a la subárea de psicología.

- LIMIT-TO (DOCTYPE, "ar" OR "re"): Incluye solo artículos de investigación o revisiones.

- LIMIT-TO (EXACTKEYWORD, "Human"): Restringe la búsqueda a estudios en humanos.

- LIMIT-TO (PUBSTAGE, "final"): Incluye solo artículos en su versión final.

- LIMIT-TO (OA, "all"): Incluye artículos de acceso abierto.

**- Results: 1.888 documentos.**

**Filter 3.2: Temporal (2010-2024)**

- Search Terms: ([cancer OR tumor]) AND [(brief OR (executive AND function))]

- Applied Filters:

- LIMIT-TO (SUBJAREA, "PSYC"): Limita la búsqueda a la subárea de psicología.

- LIMIT-TO (DOCTYPE, "ar" OR "re"): Incluye solo artículos de investigación o revisiones.

- LIMIT-TO (EXACTKEYWORD, "Human"): Restringe la búsqueda a estudios en humanos.

- LIMIT-TO (PUBSTAGE, "final"): Incluye solo artículos en su versión final.

- LIMIT-TO (OA, "all"): Incluye artículos de acceso abierto.

**- Results: 1.654 documentos.**

**Filter 3.3: Limitado a Journals**

- Search Terms: ([cancer OR tumor]) AND [(brief OR (executive AND function))]

- Applied Filters:

- PUBYEAR > 2009 AND PUBYEAR < 2025: Limita los Results a estudios publicados entre 2010 y 2024.

- LIMIT-TO (SUBJAREA, "psyc"): Limita la búsqueda a la subárea de psicología.

- LIMIT-TO (DOCTYPE, "ar" OR "re"): Incluye solo artículos de investigación o revisiones.

- LIMIT-TO (EXACTKEYWORD, "human"): Restringe la búsqueda a estudios en humanos.

- LIMIT-TO (PUBSTAGE, "final"): Incluye solo artículos en su versión final.

- LIMIT-TO (OA, "all"): Incluye artículos de acceso abierto.

- LIMIT-TO (SRCTYPE, "j"): Limita la búsqueda a artículos publicados en revistas.

**- Results: 1.650 documentos.**

**Filter 3.4: Limitado a Niños**

- Search Terms: ([cancer OR tumor]) AND [(brief OR (executive AND function))]

- Applied Filters:

- PUBYEAR > 2009 AND PUBYEAR < 2025: Limita los Results a estudios publicados entre 2010 y 2024.

- LIMIT-TO (SUBJAREA, "psyc"): Limita la búsqueda a la subárea de psicología.

- LIMIT-TO (DOCTYPE, "ar" OR "re"): Incluye solo artículos de investigación o revisiones.

- LIMIT-TO (EXACTKEYWORD, "child, preschool"): Restringe la búsqueda a estudios que incluyan niños en edad preescolar y niños.

- LIMIT-TO (PUBSTAGE, "final"): Incluye solo artículos en su versión final.

- LIMIT-TO (OA, "all"): Incluye artículos de acceso abierto.

- LIMIT-TO (SRCTYPE, "j"): Limita la búsqueda a artículos publicados en revistas.

**- Results:57 documentos.**

**Justification of the Filters**

The applied filters ensure that the results consist specifically of research studies relevant to psychology and related to executive functions in children affected by cancer or tumors, published in recent years and in academic journals. This provides a more precise and useful selection of documents for review and analysis in this specific field.

In addition to adapting the syntax to the requirements of each database, specific filters were applied in each database to facilitate the search according to the established inclusion and exclusion criteria.
