# Supplementary material for: A Systematic Review and Meta-Analysis of Executive Function Outcomes in Pediatric Central Nervous System Tumor Survivors
Source: Actas Esp Psiquiatr. 2026 Feb 15;54(1):237–62. doi: 10.62641/aep.v54i1.2053 (PMC12946733; doi:10.62641/aep.v54i1.2053)
Supplement: Supplementary file 1 [file ActEsp-54-1-237-262-s1.zip › Supplementary Material 2_Included+Studies.docx]

**Supplementary Material 2: Characteristics of the Included Studies**

The participants were under 18 years old at the time of diagnosis and/or evaluation. They were diagnosed with or were survivors of: pediatric or childhood cancer, low-grade cerebellar gliomas, neurofibromatosis type I, pediatric craniopharyngioma, or acute lymphoblastic leukemia.

The research designs of the analyzed studies were mainly comparative-causal and predictive, although not exclusively, as three meta-analyses were also included.

Executive functioning was assessed using the BRIEF scales in their different versions (BRIEF-P, BRIEF, and BRIEF-A) and modalities (self-report, parent-report, and teacher-report).

Five studies explicitly mention the use of this executive function assessment scale in their titles.

| **Nº** | **Study** | **Journal/Oncology Specific** | **Title (BRIEF)** | **Country** | **Sample**  **(n)** | **Cáncer** | **ge (diagnosis and/or evaluation)** | **Sex N(DT)** | **Methodology** | **Design** | **Instrument** | **Results** | **Overall Results** | **Meta-analysis** |
| --- | --- | --- | --- | --- | --- | --- | --- | --- | --- | --- | --- | --- | --- | --- |
|  | [1] | Journal of Clinical Oncoloy /SÍ | NO | Memphis (EE.UU) | N=139 | Brain tumor:  Medulloblastoma, all types: 41 (29.5%)  Anaplastic ependymoma: 25 (18.0%)  Atypical teratoid rhabdoid tumor: 14 (10.1%)  Ependymoma: 8 (5.8%)  Primitive neuroectodermal tumor: 11 (7.9%)  High-grade glioma, NOS: 7 (5.0%)  Others: 33 (23.7%) | Age at diagnosis: 1.7 (1.0)  (Infants recently diagnosed with a tumor) | Females: 57 (41%)  Males: 82 (59%) | IV:  > Type of treatment: chemotherapy, photon radiotherapy, or proton radiotherapy  Longitudinal evolution: 3 time points – Baseline, 6 months, 1 year, and 5 years.  DV:  > Working memory  > Global EF (BRIEF)  > Attention problems  > Adaptive behavior | Predictor | BRIEF-P  Longitudinal  Prospective | Predictive capacity of cognitive performance | Even before adjuvant therapy, young children with brain tumors experience cognitive difficulties that can affect their quality of life. Changes in cognitive functioning over time depended on the location of the tumor and surgical factors rather than adjuvant therapy. | NO |
|  | [2] | BMC Psychology / NO | YES | Cape Town (Sur África) | N=45 | Childhood cancer survivors:  Leukemia (58%)  Lymphoma (9%)  Central nervous system tumor (20%)  Other tumors (13%) | Age at diagnosis: 7.1 (4.4)  Age at evaluation: 14.0 (3.0) | Males: 32 (71.1%)  Females: 13 (28.9%) | IV:  BRIEF  CogSatet  DV:  CT Mastery Test Reading  CT Mastery Test Mathematics | It is a diagnostic validation study.  Screening methods | BRIEF  Metacognitive index  Predictive of performance | Specificity and sensitivity of BRIEF-MCI in identifying academic difficulties in survivors. | It will be important to have an easy and quick-to-administer monitoring tool, especially in communities with limited resources, to identify patients who need a full neurocognitive evaluation. Continuous effort is required to find screening measures that have both strong sensitivity and specificity. | NO |
|  | [3] | Journal of Clinical and Experimental Neuropsychology / NO | NO | France | G. NF1:  (n=33)  G. Control:  (n=52)  Informants:  Parents  (n=31)  Teachers  (n=18) | Neurofibromatosis type 1 (NF1) | 3-5 years  G. NF1:  56.67 (11.27) (months)  G. Control:  55.75 (10.37) (months) | G. NF1:  17/16 (male/female)  G. Control:  27/25 (male/female) | IV: G. NF1 vs. G. Control  DV: BRIEF-P  Intellectual competence (WPPSI-IV) | Comparative-causal | BRIEF-P  (parents and teachers) | Parents:  Flexibility, Inhibition  Teachers:  Global, Inhibition, and Emotional Control | > Early executive dysfunction in children with NF1 is supported, highlighting the need for early and systematic evaluation of executive functions (EF).  > Both performance-based tests and questionnaires are complementary tools for investigating early EF dysfunction in children with NF1. | YES  Informant:  Parents  Teachers |
|  | [4] | BMC Cancer / SÍ | NO | Switzerland | G. Pediatric cancer survivors  (n=150)  G. Control group  (n=50) | Pediatric cancer survivors | 7-16 years | Not available | IV: Experimental group vs. Control group  DV: Neuropsychological assessment, physical evaluation, questionnaires, and neuroimaging | Comparative-causal:  > Training: cognitive, physical, and control  > Measures: before and after 8 weeks of training and 3 months of follow-up | BRIEF | Intervention / cognitive and physical training in survivors. | Global executive function improves with early intervention after oncological treatment. | NO |
|  | [5] | Neuro-Oncology / SI | NO | Londres | G. Cerebellar  (N=72)  G. Medulloblastoma  (n=37)  G. Astrocytoma  (n=35)  G. Control  (n=38) | Brain tumor | 8-14 years  G. Cerebellar  N:  Medulloblastoma  Age at evaluation: 10.2 (8-14)  Age at diagnosis: 10.4 (8-14)  Astrocytoma  Age at evaluation: 10.4 (8-14)  Age at diagnosis: 9.2 (5-14)  G. Control  Age at evaluation: 10.4 (8-14) | G. Cerebellar  G. Medulloblastoma  Females: 13 (41%)  G. Astrocytoma  Females: 23 (68%)  G. Control  Females: 19 (50%) | IV:  G. Cerebellar  G. Medulloblastoma  G. Astrocytoma  G. Control  DV:  IQ  BRIEF (Parents and Teachers)  SDQ (Parents, Teachers, and Child)  PedsQL (Parents and Child) | Comparative-causal | BRIEF  Parents  Teachers | Screening  Discrimination of cognitive deficits in the educational context | The PedsQL reported by children and parents, as well as the BRIEF and SDQ reported by teachers, have moderately good accuracy in discriminating between children with and without an FSIQ lower than 80. | YES  Informants:  Parents  Teachers |
|  | [6] | BMC Medicine /NO | NO | Norway | G. Brain tumor (n=29)  G. Traumatic brain injury (n=18)  G. Stroke (n=17)  G. Infection/Inflammation (n=7)  G. Hypoxia/Anoxia (n=5) | Several | 8.5 (5-11) | Women  43 (57%) | IV:  Types of intervention: pGMT and pBHW  DV:  Cognitive functioning  Executive functioning (BRIEF) | Comparative-causal | BRIEF  Parents  Teachers  Informants | Intervention study with 2 intervention groups  Different measures: baseline, 5 weeks, 8 weeks, and 6 months | In pABI, metacognitive training (pGMT) did not show additional effectiveness in parent-reported executive function at the 6-month follow-up assessment compared to a psychoeducational control. Both interventions were well tolerated and demonstrated distinct improvements at different levels of executive function assessment. | SI  Informants: self-report, parents, teachers |
|  | [7] | Brain Inj / NO | NO | Richmond, Virginia, USA | G. TBI (Traumatic Brain Injury)  (n=102)  G. Informant  (n=39) | Primary Brain Tumor (PBT) | PBT =  49.0 (14.8)  Range: (21-81) | PBT =  Male: 54 (52.9%)  Female: 48 (47.1%) | VI: Pacientes, Informantes  VD: Neurocognitivas Psicológicas Funcionales | Comparative-causal | BRIEF-A | Global executive dysfunction | Patients and informants agreed on their reports of executive functions (EF). These reports, although not differing in neurocognitive classification, did differ in terms of psychological functioning and in those requiring assistance with instrumental activities of daily living (IADLs). Reports on EF provided by patients and informants may offer valuable data on psychological functioning and IADLs in this population. | YES  Informants:  Self-report and other informants |
|  | [8] | Magnetic Resonance Imaging / NO | NO | New York (EE.UU) | G. Pediatric cancer  (n=15)  G. Control  (n=15) | Pediatric cancer | G. Pediatric cancer:  15.12 (5.98)  Age at diagnosis:  8.31 (6)  Chemotherapy duration:  1.73 (1.12)  Post-chemotherapy time:  5.18 (5.9)  G. Control:  15.13 (4.21) | G. Pediatric cancer:  Males: 6  G. Control:  Males: 7 | IV:  Morphometry, cortical thickness, subcortical volumes using magnetic resonance imaging (MRI)  DV:  BRIEF, pegboard, and working memory assessment | Comparative-causal | BRIEF | Negative impacts of chemotherapy in pediatric patients treated during the formative years of brain development. | The neurotoxicity of systemic chemotherapy has widespread negative effects on brain development in pediatric oncology patients, with relatively mild cognitive deficits. | NO |
|  | [9] | Journal of Pediatric Psychology / NO | NO | Wisconsin (EE.UU) | G. NF1 (Neurofibromatosis type 1)  (n=26)  G. Control  (n=37) | Neurofibromatosis Type 1 (NF1) | NF1:  4.53 (0.87)  G. Control:  4.51 (0.89) | NF1  Males: 17 (65%)  Females: 9 (34%)  G. Control  Males: 23 (62%)  Females: 14 (38%) | IV:  G. NF1 vs.  G. Control  DV:  Executive functioning (BRIEF-P) | Comparative-causal | BRIEF-P | Executive functioning in NF1 | Dysfunction compared to the normative mean on the Working Memory (WM) scale and the Emerging Metacognition Index (EMI). | YES  Informant: Parents |
|  | [10] | Neuroscience Biobehavioral Reviews / NO | NO | USA | N = 23  (studies) | Does not apply | Does not apply | Does not apply | Does not apply | Systematic review | BRIEF | Long-term cognitive impairment resulting from chemotherapy treatment | Survivors who received chemotherapy treatment still experienced apparent cognitive decline, particularly in the domains of attention and executive functions. The late neurotoxic effect of methotrexate on the neurocognitive performance of survivors may be related to the dosage. | NO  (Systematic review) |
|  | [11] | Tesis doctoral | NO | USA | N=166 | Pediatric brain tumor | Age at evaluation:  9 (3.10)  Age at diagnosis:  9.03 (3.25) | Males: 102 (61.45%)  Females: 64 (38.55%) | IV/DV:  Intelligence, executive functions (EF), academic performance, attention, family environment, coping //  Child Behavior Checklist (CBCL)  Behavioral Rating Inventory of Executive Function (BRIEF)  Woodcock Johnson III Tests of Cognitive Abilities (WJ-III COG) | Predictive | BRIEF  and psychosocial outcomes | Neuropsychological profile of patients with brain tumors | Binary and multinomial logistic regression showed that changes in executive function (EF) over time are predictive of the psychosocial trajectory. The findings suggest that a subset of pediatric brain tumor survivors experience psychosocial difficulties secondary to the illness and treatment, which persist without intervention. | NO |
|  | [12] | Applied Neuropsychology: Child / NO | NO | Canada | G. PBTs:  (N=91)  Low-grade glioma (42.9%), Medulloblastoma (20.9%),  Ependymoma (11%), Craniopharyngioma (7.7%)  Other (17.6%) | Pediatric brain tumor survivors (PBTs) | PBTs:  Age at evaluation:  11.21 (2.8)  Age at diagnosis:  5.00 (3.04) | Females:  44 (48.4%) | IV/DV:  Executive Functions (BRIEF)  Social Skills (Social Skills Rating System (SSRS))  Social Adjustment (Behavior System for Children (BASC-2)) | Predictive | BRIEF  Parents | Interrelationship between executive functions (EF) and social competence | The time since diagnosis and the lowest total social skills reported by parents predicted greater social withdrawal. | NO |
|  | [13] | Qual Life Res. / NO | NO | The Netherlands | G. PBTs (n=82)  G. Healthy siblings (n=43)  G. Non-PBTs (n=45) | Pediatric brain tumor survivors (PBTs) | G. PBTs:  Age at evaluation: 13.85 (3.15)  Age at diagnosis: 6.87 (3.77)  G. Control:  Age at evaluation: 14.27 (2.44)  G. Non-PBTs:  Age at evaluation: 14.28 (3.04)  Age at diagnosis: 8.23 (3.95) | G. PBTs:  Males (H): 40 (49%)  G. Healthy siblings:  Males (H): 17 (40%)  G. Non-PBTs:  Males (H): 26 (58%) | IV:  Groups:  SPTC (Survivors of Pediatric Brain Tumors)  Healthy siblings  Informants:  Self-report vs. Parents vs. Teachers  DV:  Self-report:  HRQOL (Health-Related Quality of Life)  Self-esteem  Psychosocial adjustment  Fatigue  Parent report:  Psychosocial adjustment  Executive functioning  Teacher report:  Psychosocial adjustment  Executive functioning | Comparative-causal | BRIEF | Consecuencias neuropsicológicas (disfunción ejecutiva global) y como se relaciona con el  Funcionamiento psicosocial | This study provides the first multidimensional insight (self-report, parent report, and teacher report) into the psychosocial functioning of pediatric brain tumor survivors (PBTs) with neurocognitive complaints reported by parents. The multidimensional approach is a strength of the study due to the symptom burden of the patients and the complexity of their social situation. PBTs showed impaired psychosocial functioning across several evaluated domains: self-reported health-related quality of life (HRQOL) and fatigue, parent-reported psychosocial adjustment and executive functions, and teacher-reported psychosocial adjustment, observed only in females. | YES  Informants: Parents  Teachers |
|  | [14] | Health and Quality of Life Outcomes / NO | NO | USA | G. Pediatric cancer survivors  (n=26)  G. Control  (n=53) | Pediatric cancer survivors | G. Pediatric cancer survivors:  12.35 (3.99)  G. Control:  9.94 (3.64) | Males:  G. Pediatric cancer survivors:  53%  G. Control:  50% | IV:  G. Pediatric cancer survivors vs. G. Control  (n=53)  DV:  Social status  Functional disability (Brief Impairment Scale – BIS)  Intellectual functioning (Reynolds Intellectual Screening Test – RIST) general  Executive functioning (BRIEF) | Predictive | BRIEF  Parents | A new measure of self-reported functional impairment and exploring the relationship between self-reported functional impairment and parent-reported functional impairment in pediatric cancer survivors compared to controls. | BIS-C appears to be an adequate screening tool for identifying self-reported functional disability in children aged 5 to 18 years, although there are some validity concerns in children aged 5 to 7 years. | NO |
|  | [15] | Journal of Neuro-Oncology / SI | NO | USA | N=104 | Pediatric craniopharyngioma  prior to proton therapy treatment | 9.84 (4.74) | Males:  50 (48.08%) | IV / DV:  Cognitive competence,  Attention domain,  Visuospatial processing domain  Instruments:  CPT-II  D-KEFS  BRIEF  CVLT  ABAS-II  BASC-S  WJ-III Ach | Predictive | BRIEF  Parents | Predictor of low cognitive performance  Investigate the impact of patient-related, disease-related, and treatment-related variables on the neurocognitive outcomes of pediatric patients with craniopharyngioma before proton therapy treatment or observation after radical resection. | Patients with craniopharyngioma show weaker neurocognitive performance compared to the normative population, as a result of the tumor, the events leading to the diagnosis, and early surgical intervention.  Systematic research on neurocognitive performance before radiation therapy is essential to assess the potential risks and benefits of new radiation therapy methods, including proton therapy. | NO |
|  | [16] | Neuropsychological Rehabilitation /NO | NO | Israel | G. NF1:  (n=29)  G. Control:  (n=27) | Neurofibromatosis Type I (NF1) | NF1:  12.3 (2.6)  G. Control:  12.4 (2.5) | NF1  Males: 8  Females: 21  G. Control  Males: 8  Females: 19 | IV:  G. NF1 vs.  G. Control  DV:  BADS-C  BRIEF-Parents  ACES-Teacher | Comparative-causal | BRIEF  Parents | Predictor of academic performance | Children with NF1 exhibit executive dysfunction, which partially explains their difficulties in academic performance. | YES  Informant: Parents |
|  | [17] | Child's Nervous System / NO | NO | USA | N=101 | Pediatric brain tumor survivors  After proton radiation therapy | Age at proton radiation therapy: 8.12 (3.95)  Age at evaluation: 14.16 (3.90) | Males:  51 (50.5%)  Females:  50 (49.5%) | IV/DV:  Psychosocial functioning  BASC-Parents  Executive functioning  BRIEF-Parents | Predictive | BRIEF  Parents | Relationship between psychosocial functioning and executive functions (EF) derived from proton radiation therapy. | Psychosocial and executive functioning were within the normal range.  Social withdrawal and metacognitive executive functioning (working memory, initiation, planning/organization) were areas of concern. | NO |
|  | [18] | Child Neuropsychology / NO | NO | Turkia | Total (N=369)  Leukemia (n=117)  Lymphoma (n=30)  Brain tumor (n=120)  Neuroblastoma (n=24)  Wilms tumor (n=27)  Ewing's sarcoma (n=24)  Osteosarcoma (n=12)  Yolk sac sarcoma (n=6)  Hepatoblastoma (n=3)  Nasopharyngeal (n=6) | Various cancers | 10.48 (4.5) | Males: 201 (54.5%)  Females: 168 (45.5%) | IV:  Child self-report cognitive functioning scale  Parent proxy-report cognitive functioning scale  DV:  BRIEF | Comparative-causal | BRIEF  Parents  Teachers | Concurrent validity | PedsQL™ CFS is a reliable, valid, and culturally adapted instrument for assessing cognitive functioning in children with cancer (Turkey). | NO |
|  | [19] | Pediatric, Blood &  Cancer / SI | NO | USA | N=177 | Leukemia (B-acute lymphoblastic leukemia, B-ALL) | Age at Diagnosis:  8.4 (5.0)  <10 years  88 (48.6%)  ≥10 years  93 (51.4%) | Males:  80 (44.2%)  Females:  101 (55.8%) | Independent Variable (IV):  > Treatment Type:  - Methotrexate-derived  - Steroids  > Age at Diagnosis:  - <10 years  - ≥10 years  Dependent Variable (DV):  - BRIEF  - CPT-II  - Tower Test  - Category Fluency  - ABS Self-Direction | Comparative-causal | BRIEF | Executive Functioning and Attention Profile | In multivariable models, participants with U.S. public insurance showed significantly greater parent-reported executive functioning (EF) difficulties than those with U.S. private or non-U.S. insurance. Additionally, participants diagnosed before the age of 10 performed significantly worse on attention measures (i.e., continuous performance task) and EF measures (i.e., verbal fluency and tower planning task). | NO |
|  | [20] | Child Neuropsychology / SÍ | NO | USA | N=61 | Leukemia (B-acute lymphoblastic leukemia, B-ALL) | Age at evaluation: 5 (0.72)  Age at diagnosis: 2.62 (1.32) | Men: 33 (54.1%)  Women: 28 (45.9%) | VI:  Tumor type: brain tumor vs. other  Therapy: on vs. off  VD:  Cognitive functioning  School readiness  Adaptive functioning reported by parents  Executive functioning | Comparative-causal | BRIEF Parents  BRIEF-P (Behavior Rating Inventory of Executive Function - Parents) | Executive functioning profile in cancer-treated individuals (cognitive and functional impairments) | The results indicate the presence of emerging weaknesses in executive functioning in young children with cancer and contribute to a growing body of literature highlighting the potential cognitive and behavioral risks associated with a cancer diagnosis in early childhood. | NO |
|  | [21] | Journal of Cancer Research and Therapeutics / SÍ | NO | China | N=9 studies | Pediatric brain tumor | 6–18 years | Not applicable | IV/DV:  Cognitive functioning of pediatric cancer survivors | Review and  Meta-analysis: working memory, attention, executive functioning, and academic and intellectual performance | 3 studies include BRIEF | Effects of neurocognitive rehabilitation interventions on cognitive functioning and intellectual performance. | Neurocognitive rehabilitation interventions improve working memory, attention, and executive function in pediatric cancer survivors in post-intervention assessment and short-term follow-up. | NO  (It’s a meta-analysis) |
|  | [22] | Applied Neuropsychology: Child / NO | NO | USA | G. Medulloblastoma  (n=36)  G. Pilocytic Astrocytoma  (n=20) | Pediatric Brain Tumor:  - Medulloblastoma  - Pilocytic Astrocytoma | Medulloblastoma  - Age at Diagnosis: 8.55 (4.34)  - Age at Evaluation: 14.07 (3.45)  Pilocytic Astrocytoma  - Age at Diagnosis: 5.40 (4.34)  - Age at Evaluation: 12.84 (2.67) | Medulloblastoma  - Males: 24 (66.7%)  - Females: 12 (33.3%)  Pilocytic Astrocytoma  - Males: 11 (55.0%)  - Females: 9 (45.0%) | IV (Independent Variable):  - Type of Cancer:  - Medulloblastoma  - Pilocytic Astrocytoma  DV (Dependent Variable):  - BRIEF | Comparative-causal | BRIEF  - Parents | Sensitivity, Discrimination | The survivors of Pan demonstrate worse executive functioning than the survivors of MB. | Yes  Informants: Parents |
|  | [23] | Supportive Care in Cancer / SÍ | NO | Philadelphia, PA, USA | 2 Phases:  - Phase 1: Focused Interview Group  (n=36)  - Phase 2: Intervention Group  (n=12) | Pediatric Brain Tumor (PBTS) | Focused Interview Group  - Caregivers: 48.53 (6.76)  - Survivors:  - Age at Evaluation: 14.47 (2.29)  - Age at Diagnosis: 6.29 (3.29)  Intervention Group  - Caregivers: 51.25 (16.81)  - Survivors:  - Age at Evaluation: 13.17 (3.10)  - Age at Diagnosis: 4.89 (2.95) | Focused Interview Group  - Caregivers:  - Males: 13 (36.1%)  - Females: 23 (63.9%)  - Survivors:  - Males: 13 (54.2%)  - Females: 11 (45.8%)  Intervention Group  - Caregivers:  - Males: 2 (16.7%)  - Females: 10 (83.3%)  - Survivors:  - Males: 7 (58.3%)  - Females: 5 (41.7%) | IV / DV:  - Family Functioning:  - General Functioning Scale from the McMaster Family Assessment Device (FAD GFS)  - Social Adjustment:  - PedsQL 4.0  - Executive Functioning:  - BRIEF  - Social Skills:  - The Social Skills Improvement System (SSIS) | Association | BRIEF  Self-Report | Health-Related Quality of Life, Social Skills, Executive Functioning, and Family Functioning | The data support associations between the survivor's social skills, executive functioning, and family functioning. | NO |
|  | [24] | Journal of the International Neuropsychological Society / NO | NO | USA | Group 1: Pediatric brain tumors treated with conformational radiotherapy (n = 50)  Group 2: Healthy siblings of brain tumor survivors (n = 40)  Group 3: Survivors of solid tumors who had not received treatment targeting the CNS (n = 40) | Pediatric brain tumor | Age at Evaluation:  - Pediatric brain tumors treated with conformational radiotherapy: 13.18 (2.88)  - Healthy siblings of brain tumor survivors: 12.91 (2.62)  - Survivors of solid tumors who had not received treatment targeting the CNS: 13.21 (3.46)  Age at Diagnosis:  - Pediatric brain tumors treated with conformational radiotherapy: 6.38 (3.43)  - Healthy siblings of brain tumor survivors: NA  - Survivors of solid tumors who had not received treatment targeting the CNS: 4.50 (4.19) | Males:  - Pediatric brain tumors treated with conformational radiotherapy: 50%  - Healthy siblings of brain tumor survivors: 50%  - Survivors of solid tumors who had not received treatment targeting the CNS: 50% | IV (Independent Variable):  - Group 1: Pediatric brain tumors treated with conformational radiotherapy  - Group 2: Healthy siblings of brain tumor survivors  - Group 3: Survivors of solid tumors who had not received treatment targeting the CNS  DV (Dependent Variable):  - Working memory based on BRIEF  - Working memory based on performance tests (WISC-IV and WAIS-III) | Comparative-Causal | BRIEF  Working Memory Index (Behavioral Regulation and Metacognition) | Working Memory Scale  (Behavioral Regulation and Metacognition)  Parent Version | BRIEF does not seem to be a good screening tool for assessing working memory (WM) among brain tumor survivors, neither on its own nor as a replacement for performance-based measures. | NO |
|  | [25] | Archives of Clinical Neuropsychology / NO | NO | USA | Group 1: Brain tumor (n=31)  Group 2: Control (n=33) | Brain tumor | Age at Diagnosis:  8.84 (4.82)  Age at Evaluation:  - Survivors: 22.47 (2.57)  - Control: 21.48 (3.09) | Group 1: Survivors  - Males: 42%  - Females: 58%  Group 2: Control  - Males: 39%  - Females: 61% | IV (Independent Variable):  - Group 1: Brain tumor vs.  - Group 2: Control  DV (Dependent Variable):  - Executive Functioning: BQSS, ROCF  - Adaptive Functioning  - Socioeconomic Status  - Intelligence | Comparative-causal | BRIEF-A | Convergent Validity with Rey-Osterrieth Complex Figure (ROCF) | The mediation analyses showed that the planning skills of the BQSS mediate the relationship between the group and community living skills. The convergent findings of the BRIEF in planning, along with the discriminant findings with BQSS Fragmentation, BRIEF Emotional Control, and the Grooved Pegboard, support planning as the specific mediator in this model. | Yes  Informants: Parents or other significant individuals |
|  | [26] | Child Neuropsychology / NO | YES | USA | N = 734  - Group 1: EPI (n = 76)  - Group 2: OTC-D (n = 42)  - Group 3: BT (n = 69)  - Group 4: NF1 (n = 53)  - Group 5: ALL (n = 51)  - Group 6: ADHD control (n = 115)  - Group 7: Control (n = 328) | Various:  Leukemia (ALL), epilepsy (EPI), neurofibromatosis type 1 (NF1), ornithine transcarbamylase deficiency (OTC-D), attention deficit hyperactivity disorder (ADHD) controls, and matched healthy controls. | 5-18 | Females:  - EPI = 47.4%  - OTC-D = 71.4%  - Brain Tumor = 55.1%  - NF1 = 58.5%  - ALL = 33.3%  - ADHD Control = 61.7%  - Control = 51.5% | DV (Dependent Variable):  - Groups:  - G. EPI  - G. OTC-D  - G. BT (Brain Tumor)  - G. NF-1  - G. ALL  - G. ADHD  - G. Control  IV (Independent Variable):  - BRIEF | Comparativo-causal | BRIEF | Working Memory  Planning  Organization | Children with specific diagnoses and an early age at diagnosis and evaluation reported greater difficulties in executive functions (working memory, as well as organizational and planning skills). | NO |
|  | [27] | Brain Injury / NO | NO | France | N=29 | Pediatric craniopharyngioma | Age at Diagnosis:  7.10 (4.1)  Follow-up:  6.2 (4.5) | Not available | IV (Independent Variable):  - Group 1: Family informants vs.  - Group 2: Self-informants  DV (Dependent Variable):  - Self-report of QoL (Kidscreen 52) and depression (MDI-C)  - Proxy-reports of QoL (Kidscreen 52), executive functioning (BRIEF), and disease impact (Hoare and Russell Questionnaire) | Comparative-causal | BRIEF | Global Executive Dysfunction  Depression  Quality of Life | Depression and the low educational level of parents were associated with lower quality of life (QoL) and higher levels of executive dysfunction. | No  (Not full text) |
|  | [28] | Neuropsychological Rehabilitation / NO | NO | France | Group 1: Pediatric brain tumor (n = 21)  Group 2: Control (n = 44) | Benign or malignant brain tumor in the frontal lobe during childhood. | 8-27 | Not available | IV (Independent Variable):  - Group 1: Control vs.  - Group 2: Pediatric Brain Tumor  DV (Dependent Variable):  - BRIEF  - BADS-C | Comparative-causal | BRIEF | Global Executive Dysfunction | The patients exhibited deficits in most, but not all, executive function (EF) measures. Most classical and ecological EF measures showed a strong correlation with intelligence quotient (IQ). This study confirms the frequency of executive function deficits in this population and highlights the usefulness of ecological EF measures, as well as some limitations of classical EF tests in children. | NO |
|  | [29] | The Journal of Pediatrics / NO | NO | Australia | Group NF1 (n=43)  Control Group (n=43) | Neurofibromatosis Type 1 (NF1) | G. NF1  40.23 (0.72) months  G. Control  40.16 (0.48) months | G. NF1  H = 32 (74%)  M = 11 (26%)  G. Control  H = 32 (74%)  M = 11 (26%) | IV (Independent Variable):  G. NF1 vs. G. Control  DV (Dependent Variables):  - BASC – II  - BRIEF-P  - CADS-P | Comparative-causal | BRIEF-P  Fuente: Padres (Parents) | Preschoolers  Cognitive and executive profile | Young children with NF1 exhibit significantly lower intellectual functioning, expressive language, and visual perception. These difficulties can be detected in preschool age and are likely to impact learning and performance during the early school years. | YES  Informant: Parents |
|  | [30] | Applied Neuropsychology: Adult / NO | YES | USA | G. MCI (n = 23)  G. ADHD-U (n = 27)  G. TBI (n = 23)  G. Control (n = 26) | Primary brain tumor | Evaluation:  49.6 (range 21–81)  Age at diagnosis:  44.9 (range 13–81) | H = 38 (51.4%)  M = 36 (48.6%) | IV (Independent Variable):  - G. MCI  - G. ADHD-U  - TBI  - G. Control  DV (Dependent Variable):  - BRIEF-A | Comparative-causal | BRIEF-A | Metacognition  Behavioral Regulation | Although the group averages did not reach levels of clinical impairment, a substantial proportion of patients with PBT showed executive dysfunction. The elevations were more prominent in metacognitive skills than in behavioral dysregulation. It is noteworthy that the executive function profile of patients with PBT was notably similar to that of the MCI and TBI groups, higher than that of the HC group, but clearly lower than that of the ADHD-U group. | NO |
|  | [31] | Applied Neuropsychology: Child / NO | NO | USA | G. Surgical resection of glioma during childhood  (n = 12)  G. Control  (n = 12) | Low-grade cerebellar gliomas | Age at surgery:  8.8 (3.0)  Age at evaluation:  13.8 (5.6) | Men: 4  Women: 8 | IV / DV:  - G. Anxiety (MASC, MAS)  - Fear of pain (FOPQ-C, FOPQ-III)  - Executive Function (BRIEF/BRIEF-A) | Comaprtive-causal | BRIEF  BRIEF-A | Long-term impact of surgical-only treatment (without exposure to other treatments, such as chemotherapy and radiation) | Pediatric survivors of cerebellar tumors treated with surgery alone have favorable long-term functioning. | NO |
|  | [32] | Child Neuropsychology / NO | NO | Australia | G. Control  (n=55)  G. NF1  (n=191)  G. NF1 Typical  (n=41)  G. NF1 Bordeline  (n=30)  G. NF1 Impaired  (n=120) | Neurofribromatosis tipo 1 | Control  11.81 (2.61)  NF1  10.38 (2.36)  NF1 Typical  11.61 (2.75)  NF1 Bordeline  9.98 (2.29)  NF1 Impaired  10.06 (2.11) | Hombres=  Control  22(40)  NF1  104(54.45)  NF1 Typical  27(65.85)  NF1 Bordeline  13(56.67)  NF1 Impaired  64(53.33) | IV: G. NF1 vs. G. Control  VD: RCFT, IQ, Visuospatial abilities, BRIEF, Tower of London, The Conners ADHD DSM-IV Scales (CDAS) | Comparative-causal | BRIEF | FE global | This study provides evidence that visuospatial deficits are a key factor in the decreased performance on the RCFT in children with NF1, and that executive skills, as well as younger age, are also independent predictors of performance on the RCFT. | YES  Informant: Parents |
|  | [33] | Pediatric  Blood &  Cancer / SI | NO | USA | N=137 | Pediatric brain tumor (survivors) | Age at diagnosis:  5.94 (3.16–8.98)  Age at neuropsychological evaluation:  12.63 (9.33–15.62) | H = 60 (43.8%)  M = 77 (56.2%) | Predictive study  IV (Independent Variable):  PedsQL (parents, informants)  DV (Dependent Variable):  BRIEF (parents and informant) | Predictive  Comparative-Causal | BRIEF  - Parents  - Informant | Cognitive regulation  Behavioral regulation  Emotional regulation  Global | Current data demonstrate that executive function (EF) is a significant predictor of health-related quality of life (HRQOL) during survival in young individuals previously diagnosed with pediatric brain tumors. There may be opportunities to intervene and improve the HRQOL of pediatric brain tumor survivors by focusing on executive function. | NO |
|  | [34] | Journal of Neuro-Oncology / SI | NO | USA | G. Brain Tumor  (n = 45)  - Ependymoma 21 (47%)  - Low-grade glioma 9 (20%)  - Craniopharyngioma 15 (33%)  G. Siblings  (n = 36)  G. Solid Tumor  (n = 33) | Brain Tumor  - G. Ependymoma (n = 21)  - Low-grade Glioma (n = 9)  - Craniopharyngioma (n = 15) | G. Brain Tumor  - Age at diagnosis: 6.11 (3.45)  - Age at evaluation: 12.67 (2.56)  G. Siblings  - Age at diagnosis: NA  - Age at evaluation: 12.37 (2.13)  G. Solid Tumor (n = 33)  - Age at diagnosis: 3.36 (2.87)  - Age at evaluation: 12.18 (2.88) | Men  - G. Brain Tumor: 46.7%  - G. Siblings: 47.2%  - G. Solid Tumor: 48.5% | IV: Groups  - G. Brain Tumor  - G. Siblings  - G. Solid Tumor  DV:  - HRQoL (survivors)  - HRQoL (parents)  - HRQoL associated with cognitive functioning | Comparative-causal | BRIEF | Association with another construct  Health-related quality of life (HRQoL) | HRQoL was associated with executive function (EF), but not with IQ. | NO |
|  | [35] | Child Neuropsychology / NO | NO | Australia | G. NF1:  (n = 168)  G. Control:  (n = 55) | Neurofibromatosis Type 1 (NF1) | 6-16  G. NF1: 10.62 (2.28)  G. Control: 11.24 (2.03) | G. NF1:  - H = 108  - M = 91  G. Control: (data missing) | IV:  G. NF1 vs. G. Control  DV:  - BRIEF  - Conners' ADHD DSM-IV Scales (CADS)  - Wechsler Intelligence Scales for Children-Third Edition or Fourth Edition (WISC-III / WISC-IV) | Comparative-causal | BRIEF  (parents and teachers) | Attention | The prevalence of functional and executive attention deficits was examined in a large sample of children with NF1, and the relationship between scores on cognitive tests and functional indices was assessed. Our results suggest that although the convergent validity of these two domains was quite low, both have the ability to detect significant impairments and contribute important information to the clinical picture of a specific child. We argue that neuropsychological evaluations should include both cognitive and functional tests to provide more accurate and sensitive information about a child's strengths and weaknesses to guide intervention programs. | SÍ |
|  | [36] | Pediatric Blood & Cancer / SI | NO | USA | N=56 | Brain tumor | Age at diagnosis:  6.94 (4.05)  Age at evaluation:  - Time 1: 9.91 (3.64)  - Time 2: 11.86 (3.60) | H = 29 (52%)  M = 27 (48%) | IV:  - Time 1  - Time 2  DV:  - Behavioral Regulation  - Emotional Regulation  - Global Executive Functioning  - Metacognition  - Working Memory | Compartive-causaL | BRIEF  BRIEF-P  BRIEF-2 | Behavioral Regulation  Metacognition  Working Memory | Multiple measures of executive functions (EF) should be considered when providing diagnoses and recommendations for pediatric brain tumor survivors. Furthermore, given the decline over time, the findings document the need for ongoing monitoring and reassessment of survivors as they move further away from treatment. | YES  Evaluation time:  Executive function from the initial neuropsychological assessment to follow-up. |
|  | [37] | Developmental Neuropsychology /NO | NO | Noruega | G. PBT:  (n = 48)  Diagnosis:  - Ependymomas and choroid plexus tumors: 6 (12.5%)  - Astrocytomas: 23 (47.9%)  - Embryonal tumors: 16 (33.3%)  - Other gliomas: 1 (2.1%)  - Other CNS tumors: 2 (4.2%)  G. Control:  (n = 73) | Primary Brain Tumors | Received treatment for primary brain tumors (PBT) at ≤16 years, (range 13 - 17). | Not available | IV1: G. Pediatric Brain Tumors vs. G. Control  IV2: G. Pediatric Brain Tumors vs. G. Control  DV:  - BRIEF  - PedsQL  - PedsQL-MFS | Comparative-causal | BRIEF | Global executive dysfunction | Survivors reported significantly more issues with adaptive functioning compared to a healthy control group, and this was more strongly associated with executive dysfunction than with psychological symptoms and fatigue. The findings have important implications for long-term follow-up. | YES |
|  | [38] | Brain Injury / NO | YES | FrancE | N=153 | Pediatric brain tumor | Age at diagnosis: 6 (3.61)  Age at evaluation: 11.79 (3.39)  Range: 5-18 years | H = 86 (56.2%)  M = 67 (43.8%) | IV:  - Age at diagnosis  - Age at evaluation  - Radiotherapy  - Parental education level  DV:  - Global executive functioning  - Behavioral regulation  - Metacognition | Predictive | BRIEF  Parents | Global executive dysfunction | Parents of children treated for brain tumors report widespread and persistent deficits in executive functions (EF) that negatively affect their daily functioning. These difficulties vary from one patient to another. Including the analysis of all clinical scales and composite indices allows for a more comprehensive approach and facilitates the specification of the patient's executive profile. This study focused on the consequences of EF deficits on daily life functioning, but a comprehensive approach to EF requires the combined use of performance-based measures and questionnaires. | NO |
|  | [39] | Pediatric Blood & Cancer / SI | NO | Canada | G. ALL  (n=38)  G. Control_Hermanos  (n=20)  G. Control  (n=38)  In English, this would be:  Group ALL  (n=38)  Group Control_Siblings  (n=20)  Group Control  (n=38) | Acute lymphoblastic leukemia without cranial radiation therapy (ALL) | ALL  11.39 (2.44)  Age at diagnosis:  4.27 (1.97)  Average treatment time:  4.83 (1.52)  Time since treatment:  4.83 (1.52)  G. Control_Siblings  13.05 (3.30)  G. Control  11.24 (2.43) | G. ALL  Men: 20 (52.6%)  Women: 18 (47.4%)  G. Control_Siblings  Men: 13 (65.0%)  Women: 7 (35.0%)  G. Control  Men: 20 (52.6%)  Women: 18 (47.4%) | VI:  G. ALL  G. Control_Siblings  G. Control  VD:  Social adjustment:  Behavioral Assessment System for Children, Third Edition (BASC-3)  Executive functioning:  Behavior Rating Inventory of Executive Functions (BRIEF-2) | Comparative-causal | BRIEF-2 Self-report. | Predictors related to social adjustment illness in survivors. Executive functioning explains the differences in social adjustment between groups and in relation to the disease-related predictors | Survivors of pediatric acute lymphoblastic leukemia are at a higher risk of social difficulties compared to non-oncological controls. Predictors of social adjustment among survivors included executive functioning and time off treatment. However, executive functioning did not explain any of the observed group differences in social adjustment | YES  Informant: Parents |
|  | [40] | Psycho‐Oncology /SI | NO | USA | N=166 | Pediatric cancer = 57.8% cancer | Age at evaluation: 11.57 (3.82)  Age at diagnosis: 6.21 (4.06) | 45.8% women | La traducción al inglés sería:  IV:  Groups  Informants  Suicidal ideation  DV:  Neuropsychological assessment (working memory, processing speed, CVLT, and TOL) | Comparative-causal | BRIEF | Executive Function and Suicidal Ideation | A high number of children treated for cancer experience suicidal ideation (SI) and related neurocognitive issues. Screening for SI and a deeper evaluation of the connection between executive functioning and SI in pediatric cancer populations is necessary. | SI  Informant: Parents and teachers |
|  | [41] | Early Human Development /NO | NO | The Netherlands | Group exposed to chemotherapy and surgery (n=37)  Control group (n=37) | Exposed to chemotherapy during the prenatal period | Chemotherapy-exposed group:  6.1 (5.3-7.0)  Control group:  6.2 (5.3-7.0) | Chemotherapy-exposed group:  Men: 16 (43.2%)  Women: 21 (56.8%)  Control group:  Men: 16 (43.2%)  Women: 21 (56.8%) | IV:  Informant  Test language  Group  DV: BRIEF | Comparative-causal | BRIEF | Emotional regulation | The overall results of executive functioning were reassuring. However, children prenatally exposed to chemotherapy have weaker emotional regulation skills compared to their matched controls. The results highlight the need for long-term follow-up for these children. | NO |
|  | [42] | Child Neuropsychology / NO | NO | Amsterdam | N=82 | Pediatric Brain Tumor Survivor (PBTS) | Age at diagnosis:  6.9 (3.8)  Age at evaluation:  13.8 (3.2) | Men: 40 (49%)  Women: 42 (51%) | IV:  Tasks:  Attention and cognitive flexibility (Attention Network Test – ANT)  Inhibition (Stop Signal Task)  Short-term visual memory (Visual Sequencing Task)  Verbal Working Memory (WISC-III / WAIS-III Digits)  DV: Questionnaires (BRIEF) | Predictive | BRIEF  Parents  BRIEF  Teachers | Correlations between BRIEF Parents, BRIEF Teachers, and traits  Correlation between BRIEF-Parents and BRIEF-Teachers  Executive Function Tasks in clinical versus non-clinical populations | Relying solely on the BRIEF as a measure of executive function evaluation in pediatric brain tumor survivors (PBTS) is insufficient. The questionnaires and tasks provide valuable and distinctive information. | YES  SI  Informants: Parents and teachers |
|  | [43] | Child Neuropsychology / NO | NO | Canada | La traducción al inglés sería:  All Group:  (n=130)  Control Group:  (n=158)  Si necesitas más ayuda, ¡avísame! | Acute Lymphoblastic Leukemia (ALL) | 8-18  Age at diagnosis  11.6 months to 11.2 years  Mean  4.1 (2.0) | ALL  Men: 78  Women: 52  Control Group  Men: 88  Women: 70 | IV:  ALL Group:  Vs.  Control Group:  DV:  Cognitive neuroscience paradigms:  Working memory (n-back task)  Inhibition: stop detection task  Task-based neuropsychological performance:  General cognitive abilities (Wechsler Intelligence Scales)  Laterality and motor speed  Mathematical abilities (WIAT-III)  Executive functions (D-KEFS, Trail Making, and Verbal Fluency)  Attention and executive deficits (parents):  Conners – 3 Parent Rating Scale and BRIEF | Comparative-causal | BRIEF  Parents | Late neurocognitive and behavioral effects | The use of cognitive neuroscience paradigms complements our understanding of the cognitive deficits evident after ALL treatment. | NO |
|  | [44] | JPediatrHematolOncol. /SI | YES | USA | Acute Lymphoblastic Leukemia Group (n=256) | Acute Lymphoblastic Leukemia (ALL) | Age at diagnosis  3.9 (1.8)  Age at evaluation  12.8 (2.5) | Men  136 (53%)  Women  120 (47%) | IV/DV:  Special education outcomes, Attention Deficit Hyperactivity Disorder (ADHD),  BRIEF | Predictive | BRIEF  Parents | Clinical utility of BRIEF  (determine if BRIEF in its parent version identifies leukemia survivors with cognitive impairment) | La traducción al inglés sería:  BRIEF completed by parents is associated with clinical outcomes, but it is not a sensitive tool for identifying leukemia survivors who require a comprehensive neuropsychological evaluation.  Si necesitas más ayuda, ¡estoy aquí para lo que necesites! | NO |
|  | [45] | Child Neuropsychology / NO | NO | USA | Acute Lymphoblastic Leukemia Group (n=256)  Control Group (n=256) | Leucemia linfoblástica aguda pediátrica de riesgo estándar (SR-ALL)  Pediatric standard risk acute lymphoblastic leukemia (SR-ALL) | Age at diagnosis  13.04 (2.43) | 55% men | IV: SR-ALL Group vs. Control Group  DV: EF (Executive Functioning) | Comparative-causal | BRIEF | Scores of the Leukemia Group above the Control Group on all scales.  Higher scores in working memory and monitoring. | Survivors were assessed as having clinical impairments in flexibility, initiation, working memory, and emotional control at rates two to three times higher than those of the HC group. The risk of deficits in working memory and self-control was higher in survivors who were older at the time of evaluation. There was no relationship between age at diagnosis or treatment regimen and executive functioning. There is a preservation of extensive and severe executive deficits in survivors of SR-ALL. However, a subset of survivors exhibits clinically significant executive dysfunction. There seems to be increased susceptibility to altered metacognitive functions as survivors age. This has implications for how we monitor the neurocognitive development and functioning of SR-ALL survivors and highlights opportunities for cognitive interventions. | NO |
|  | [46] | Cancers | NO | China | N= 50 estudios | Brain Tumor Survivors | 2-23 years | Not applicable | IV/DV:  Cognitive functioning of pediatric cancer survivors | Review and Meta-analysis: Psychosocial, Emotion, and Attention | 3 studies include BRIEF | Systematically analyze the rates of emotional, psychosocial, and attention problems in pediatric brain tumor survivors. | Pediatric Brain Tumor Survivors (PBTS) have an elevated risk of neurobehavioral impairment. | NO  (It is a meta-analysis) |
|  | [47] | The Clinical Neuropsychologist / NO | NO | USA | Brain tumor survivors group  (n=62)  Informants:  Parents:  (n=62)  Teachers:  (n=50)  Medulloblastoma 18 (29.0%)  Ependymoma 10 (16.1%)  Astrocytoma 9 (15.5%)  Glioma 7 (11.3%)  Germinoma 7 (11.3%)  Craniopharyngioma 2 (3.2%)  Other tumor types 9 (14.5%)  Control group  (n=62) | Pediatric Brain Tumor Survivor (PBTS) | Brain tumor survivors group  12.02 (3.56)  Control group  11.79 (3.21) | BT  M: (30, 48.6%)  F: (32, 51.6%)  Control group  M: (31, 50%)  F: (31, 50%) | IV: Brain tumor group  vs. Control group  DV: BRIEF | Comparative-causal | BRIEF | Working memory;  Cognitive/behavioral flexibility;  Emotional regulation;  Self-initiation;  Initiation;  Working memory;  Planning and organization | In general, teacher ratings of executive function (EF) impairment in pediatric brain tumor survivors were significantly higher than those of parents, who reported far fewer EF problems. Possible explanations for the discrepancies between evaluators include potential reporting bias/shift in parent responses and/or differences in EF demands across various contexts. | Yes  Informants: Parents and teachers |
|  | [48] | Child Neuropsychology /NO | NO | USA | N=24 | Pediatric Brain Tumor Survivor (PBTS) | 9.1(3.67) | 11 de 24 (46%) | IV/DV: Executive functions, Social skills  IQ | Predictive | BRIEF | Global executive dysfunction  Computerized EF task  Executive skills in real life | Social functioning was related to a specific aspect of executive functions, namely, the variability in response time of the survivors, such that inconsistent responses were associated with better social skills reported by both parents and the survivors themselves, regardless of intellectual abilities. Additionally, global executive skills in real life reported by parents predicted the social skills reported by parents. | NO |

ADHD-U = Unmedicated Attention Deficit Hyperactivity Disorder

ALL = Leukemia

BT = Brain Tumor

EPI = Epilepsy

H = Male.

MCI = Mild Cognitive Impairment

M = Female

NF1 = Neurofibromatosis Type 1

OTC-D = Ornithine Transcarbamylase Deficiency

PBT = Pediatric Brain Tumors

STCP = Pediatric Brain Tumor Survivor

TBI = Traumatic Brain Injury

TCP = Primary Brain Tumors

**REFERENCIAS BIBLIOGRÁFICAS**

[1] Ali JS, Ashford JM, Swain MA, Harder LL, Carlson-Green BL, Miller JM, *et al*. Predictors of Cognitive Performance Among Infants Treated for Brain Tumors: Findings From a Multisite, Prospective, Longitudinal Trial. Journal of Clinical Oncology: Official Journal of the American Society of Clinical Oncology. 2021; 39: 2350–2358. https://doi.org/10.1200/JCO.20.01687.

[2] Balsamo LM, Mitchell HR, Ross W, Metayer C, Hardy KK, Kadan-Lottick NS. Monitoring neurocognitive functioning in childhood cancer survivors: evaluation of CogState computerized assessment and the Behavior Rating Inventory of Executive Function (BRIEF). BMC Psychology. 2019; 7: 26. https://doi.org/10.1186/s40359-019-0302-3.

[3] Beaussart-Corbat ML, Barbarot S, Farges D, Martin L, Roy A. Executive functions in preschool-aged children with neurofibromatosis type 1: Value for early assessment. Journal of Clinical and Experimental Neuropsychology. 2021; 43: 163–175. https://doi.org/10.1080/13803395.2021.1893277.

[4] Benzing V, Eggenberger N, Spitzhüttl J, Siegwart V, Pastore-Wapp M, Kiefer C, *et al*. The Brainfit study: efficacy of cognitive training and exergaming in pediatric cancer survivors - a randomized controlled trial. BMC Cancer. 2018; 18: 18. https://doi.org/10.1186/s12885-017-3933-x.

[5] Bull KS, Liossi C, Peacock JL, Yuen HM, Kennedy CR, Children's Cancer and Leukaemia Group (CCLG). Screening for cognitive deficits in 8 to 14-year old children with cerebellar tumors using self-report measures of executive and behavioral functioning and health-related quality of life. Neuro-oncology. 2015; 17: 1628–1636. https://doi.org/10.1093/neuonc/nov129.

[6] Brandt AE, Finnanger TG, Hypher RE, Rø TB, Skovlund E, Andersson S, *et al*. Rehabilitation of executive function in chronic paediatric brain injury: a randomized controlled trial. BMC Medicine. 2021; 19: 253. https://doi.org/10.1186/s12916-021-02129-8.

[7] Braun SE, Lanoye A, Aslanzadeh FJ, Loughan AR. Subjective executive dysfunction in patients with primary brain tumors and their informants: relationships with neurocognitive, psychological, and daily functioning. Brain Injury. 2021; 35: 1665–1673. https://doi.org/10.1080/02699052.2021.2008492.

[8] Cahaney C, Stefancin P, Coulehan K, Parker RI, Preston T, Goldstein J, *et al*. Anatomical brain MRI study of pediatric cancer survivors treated with chemotherapy: Correlation with behavioral measures. Magnetic Resonance Imaging. 2020; 72: 8–13. https://doi.org/10.1016/j.mri.2020.05.007.

[9] Casnar CL, Klein-Tasman BP. Parent and Teacher Perspectives on Emerging Executive Functioning in Preschoolers With Neurofibromatosis Type 1: Comparison to Unaffected Children and Lab-Based Measures. Journal of Pediatric Psychology. 2017; 42: 198–207. https://doi.org/10.1093/jpepsy/jsw042.

[10] Cheung YT, Krull KR. Neurocognitive outcomes in long-term survivors of childhood acute lymphoblastic leukemia treated on contemporary treatment protocols: A systematic review. Neuroscience and Biobehavioral Reviews. 2015; 53: 108–120. https://doi.org/10.1016/j.neubiorev.2015.03.016.

[11] Cox LE. Survivors Of Pediatric Brain Tumors: Psychosocial Outcomes And Executive Function Doctoral dissertation. University of Mississippi: USA. 2016.

[12] Desjardins L, Solomon A, Janzen L, Bartels U, Schulte F, Chung J, *et al*. Executive functions and social skills in pediatric brain tumor survivors. Applied Neuropsychology. Child. 2020; 9: 83–91. https://doi.org/10.1080/21622965.2018.1522589.

[13] de Ruiter MA, Schouten-van Meeteren AYN, van Vuurden DG, Maurice-Stam H, Gidding C, Beek LR, *et al*. Psychosocial profile of pediatric brain tumor survivors with neurocognitive complaints. Quality of Life Research: an International Journal of Quality of Life Aspects of Treatment, Care and Rehabilitation. 2016; 25: 435–446. https://doi.org/10.1007/s11136-015-1091-7.

[14] Erickson SJ, Hile S, Kubinec N, Annett RD. Self-reported and parent proxy reported functional impairment among pediatric cancer survivors and controls. Health and Quality of Life Outcomes. 2020; 18: 142. https://doi.org/10.1186/s12955-020-01387-z.

[15] Fournier-Goodnight AS, Ashford JM, Merchant TE, Boop FA, Indelicato DJ, Wang L, *et al*. Neurocognitive functioning in pediatric craniopharyngioma: performance before treatment with proton therapy. Journal of Neuro-oncology. 2017; 134: 97–105. https://doi.org/10.1007/s11060-017-2492-y.

[16] Gilboa Y, Rosenblum S, Fattal-Valevski A, Toledano-Alhadef H, Josman N. Is there a relationship between executive functions and academic success in children with neurofibromatosis type 1? Neuropsychological Rehabilitation. 2014; 24: 918–935. https://doi.org/10.1080/09602011.2014.920262.

[17] Grieco JA, Evans CL, Yock TI, Pulsifer MB. Psychosocial and executive functioning late effects in pediatric brain tumor survivors after proton radiation. Child's Nervous System: ChNS: Official Journal of the International Society for Pediatric Neurosurgery. 2024; 40: 3553–3561. https://doi.org/10.1007/s00381-024-06579-2.

[18] Güney Yılmaz G, Tanrıverdi M, Şahin S, Çakır FB. Cross-cultural adaptation, reliability, and validity of the Turkish Pediatric Quality of Life Inventory-Cognitive Functioning Scale (PedsQL^TM^-CFS) in children with cancer. Child Neuropsychology: a Journal on Normal and Abnormal Development in Childhood and Adolescence. 2025; 31: 278–292. https://doi.org/10.1080/09297049.2024.2364205.

[19] Hardy KK, Embry L, Kairalla JA, Sharkey C, Gioia AR, Griffin D, *et al*. Attention and executive functioning in children and adolescents treated for high-risk acute lymphoblastic leukemia: A report from the Children's Oncology Group (COG). Pediatric Blood & Cancer. 2024; 71: e31179. https://doi.org/10.1002/pbc.31179.

[20] Harman JL, Molnar AE, Jr, Cox LE, Jurbergs N, Russell KM, Wise J, *et al*. Parent-reported executive functioning in young children treated for cancer. Child Neuropsychology: a Journal on Normal and Abnormal Development in Childhood and Adolescence. 2019; 25: 548–560. https://doi.org/10.1080/09297049.2018.1503647.

[21] He F, Huang H, Ye L, Wen X, Cheng ASK. Meta-analysis of neurocognitive rehabilitation for cognitive dysfunction among pediatric cancer survivors. Journal of Cancer Research and Therapeutics. 2022; 18: 2058–2065. https://doi.org/10.4103/jcrt.jcrt_1429_22.

[22] Holland AA, Shamji JF, Clem MA, Perez R, Palka JM, Stavinoha PL. Parent ratings of executive functioning in pediatric survivors of medulloblastoma and pilocytic astrocytoma. Applied Neuropsychology. Child. 2024; 13: 52–61. https://doi.org/10.1080/21622965.2022.2123707.

[23] Hocking MC, Quast LF, Brodsky C, Deatrick JA. Caregiver perspectives on the social competence of pediatric brain tumor survivors. Supportive Care in Cancer: Official Journal of the Multinational Association of Supportive Care in Cancer. 2017; 25: 3749–3757. https://doi.org/10.1007/s00520-017-3805-6.

[24] Howarth RA, Ashford JM, Merchant TE, Ogg RJ, Santana V, Wu S, *et al*. The utility of parent report in the assessment of working memory among childhood brain tumor survivors. Journal of the International Neuropsychological Society: JINS. 2013; 19: 380–389. https://doi.org/10.1017/S1355617712001567.

[25] King TZ, Smith KM, Ivanisevic M. The Mediating Role of Visuospatial Planning Skills on Adaptive Function Among Young-Adult Survivors of Childhood Brain Tumor. Archives of Clinical Neuropsychology: the Official Journal of the National Academy of Neuropsychologists. 2015; 30: 394–403. https://doi.org/10.1093/arclin/acv033.

[26] Krivitzky LS, Walsh KS, Fisher EL, Berl MM. Executive functioning profiles from the BRIEF across pediatric medical disorders: Age and diagnosis factors. Child Neuropsychology: a Journal on Normal and Abnormal Development in Childhood and Adolescence. 2016; 22: 870–888. https://doi.org/10.1080/09297049.2015.1054272.

[27] Laffond C, Dellatolas G, Alapetite C, Puget S, Grill J, Habrand JL, *et al*. Quality-of-life, mood and executive functioning after childhood craniopharyngioma treated with surgery and proton beam therapy. Brain Injury. 2012; 26: 270–281. https://doi.org/10.3109/02699052.2011.648709.

[28] Longaud-Valès A, Chevignard M, Dufour C, Grill J, Puget S, Sainte-Rose C, *et al*. Assessment of executive functioning in children and young adults treated for frontal lobe tumours using ecologically valid tests. Neuropsychological Rehabilitation. 2016; 26: 558–583. https://doi.org/10.1080/09602011.2015.1048253.

[29] Lorenzo J, Barton B, Arnold SS, North KN. Cognitive features that distinguish preschool-age children with neurofibromatosis type 1 from their peers: a matched case-control study. The Journal of Pediatrics. 2013; 163: 1479–1483.e1. https://doi.org/10.1016/j.jpeds.2013.06.038.

[30] Loughan AR, Braun SE, Lanoye A. Executive dysfunction in neuro-oncology: Behavior Rating Inventory of Executive Function in adult primary brain tumor patients. Applied Neuropsychology. Adult. 2020; 27: 393–402. https://doi.org/10.1080/23279095.2018.1553175.

[31] Lunde CE, Dudek MR, Talbert CA, Sieberg CB, Silva KE, Papadelis C, *et al*. The long-term impact of cerebellar tumor resection on executive functioning, anxiety, and fear of pain: A mixed methodology pilot study. Applied Neuropsychology. Child. 2025; 14(4): 496–506. https://doi.org/10.1080/21622965.2024.2337208.

[32] Maier A, Pride NA, Hearps SJC, Shah N, Porter M, North KN, *et al*. Neuropsychological factors associated with performance on the rey-osterrieth complex figure test in children with neurofibromatosis type 1. Child Neuropsychology: a Journal on Normal and Abnormal Development in Childhood and Adolescence. 2024; 30: 348–359. https://doi.org/10.1080/09297049.2023.2199975.

[33] Murphy C, Upshaw NC, Thomas AS, Fong G, Janss A, Mazewski C, *et al*. Impact of executive functioning on health-related quality of life of pediatric brain tumor survivors. Pediatric Blood & Cancer. 2021; 68: e29130. https://doi.org/10.1002/pbc.29130.

[34] Netson KL, Ashford JM, Skinner T, Carty L, Wu S, Merchant TE, *et al*. Executive dysfunction is associated with poorer health-related quality of life in pediatric brain tumor survivors. Journal of Neuro-oncology. 2016; 128: 313–321. https://doi.org/10.1007/s11060-016-2113-1.

[35] Payne JM, Hyman SL, Shores EA, North KN. Assessment of executive function and attention in children with neurofibromatosis type 1: relationships between cognitive measures and real-world behavior. Child Neuropsychology: a Journal on Normal and Abnormal Development in Childhood and Adolescence. 2011; 17: 313–329. https://doi.org/10.1080/09297049.2010.542746.

[36] Peterson RK, Jacobson LA. Changes in executive function in pediatric brain tumor survivors. Pediatric Blood & Cancer. 2022; 69: e29483. https://doi.org/10.1002/pbc.29483.

[37] Puhr A, Ruud E, Anderson V, Due-Tønnessen BJ, Skarbø AB, Finset A, *et al*. Executive Function and Psychosocial Adjustment in Adolescent Survivors of Pediatric Brain Tumor. Developmental Neuropsychology. 2021; 46: 149–168. https://doi.org/10.1080/87565641.2021.1900191.

[38] Roche J, Câmara-Costa H, Roulin JL, Chevignard M, Frappaz D, Guichardet K, *et al*. Assessment of everyday executive functioning using the BRIEF in children and adolescents treated for brain tumor. Brain Injury. 2020; 34: 583–590. https://doi.org/10.1080/02699052.2020.1725982.

[39] Schulte FSM, Merz EL, Russell KB, Tromburg C, Cho S, Tran A, *et al*. Social adjustment in survivors of acute lymphoblastic leukemia without cranial radiation therapy. Pediatric Blood & Cancer. 2022; 69: e29407. https://doi.org/10.1002/pbc.29407.

[40] Sharkey CM, Hardy KK, Gioia A, Weisman H, Walsh K. Suicidal ideation and executive functioning in pediatric cancer. Psycho-oncology. 2022; 31: 745–752. https://doi.org/10.1002/pon.5858.

[41] van Gerwen M, Vandenbroucke T, Gorissen AS, van Grotel M, van den Heuvel-Eibrink M, Verwaaijen E, *et al*. Executive functioning in 6 year old children exposed to chemotherapy in utero. Early Human Development. 2020; 151: 105198. https://doi.org/10.1016/j.earlhumdev.2020.105198.

[42] de Vries M, de Ruiter MA, Oostrom KJ, Schouten-Van Meeteren AYN, Maurice-Stam H, Oosterlaan J, *et al*. The association between the behavior rating inventory of executive functioning and cognitive testing in children diagnosed with a brain tumor. Child Neuropsychology: a Journal on Normal and Abnormal Development in Childhood and Adolescence. 2018; 24: 844–858. https://doi.org/10.1080/09297049.2017.1350262.

[43] Van Der Plas E, Erdman L, Nieman BJ, Weksberg R, Butcher DT, O'connor DL, *et al*. Characterizing neurocognitive late effects in childhood leukemia survivors using a combination of neuropsychological and cognitive neuroscience measures. Child Neuropsychology: a Journal on Normal and Abnormal Development in Childhood and Adolescence. 2018; 24: 999–1014. https://doi.org/10.1080/09297049.2017.1386170.

[44] Viola A, Balsamo L, Neglia JP, Brouwers P, Ma X, Kadan-Lottick NS. The Behavior Rating Inventory of Executive Function (BRIEF) to Identify Pediatric Acute Lymphoblastic Leukemia (ALL) Survivors At Risk for Neurocognitive Impairment. Journal of Pediatric Hematology/oncology. 2017; 39: 174–178. https://doi.org/10.1097/MPH.0000000000000761.

[45] Walsh KS, Paltin I, Gioia GA, Isquith P, Kadan-Lottick NS, Neglia JP, *et al*. Everyday executive function in standard-risk acute lymphoblastic leukemia survivors. Child Neuropsychology: a Journal on Normal and Abnormal Development in Childhood and Adolescence. 2015; 21: 78–89. https://doi.org/10.1080/09297049.2013.876491.

[46] Wang Y, Liu APY, Lee TMC, Wong WHS, Fong DYT, Leung LK, *et al*. Neurobehavioral Impairment in Pediatric Brain Tumor Survivors: A Meta-Analysis. Cancers. 2022; 14: 3269. https://doi.org/10.3390/cancers14133269.

[47] Wochos GC, Semerjian CH, Walsh KS. Differences in parent and teacher rating of everyday executive function in pediatric brain tumor survivors. The Clinical Neuropsychologist. 2014; 28: 1243–1257. https://doi.org/10.1080/13854046.2014.971875.

[48] Wolfe KR, Walsh KS, Reynolds NC, Mitchell F, Reddy AT, Paltin I, *et al*. Executive functions and social skills in survivors of pediatric brain tumor. Child Neuropsychology: a Journal on Normal and Abnormal Development in Childhood and Adolescence. 2013; 19: 370–384. https://doi.org/10.1080/09297049.2012.669470.
